# Glycogen controls *Caenorhabditis elegans* lifespan and resistance to oxidative stress

**DOI:** 10.1038/ncomms15868

**Published:** 2017-06-19

**Authors:** Ivan Gusarov, Bibhusita Pani, Laurent Gautier, Olga Smolentseva, Svetlana Eremina, Ilya Shamovsky, Olga Katkova-Zhukotskaya, Alexander Mironov, Evgeny Nudler

**Affiliations:** 1 Department of Biochemistry and Molecular Pharmacology, New York University School of Medicine, New York, New York 10016, USA; 2 Engelhardt Institute of Molecular Biology, Russian Academy of Science, Moscow 119991, Russia; 3 Howard Hughes Medical Institute, New York University School of Medicine, New York, New York 10016, USA

## Abstract

A high-sugar diet has been associated with reduced lifespan in organisms ranging from worms to mammals. However, the mechanisms underlying the harmful effects of glucose are poorly understood. Here we establish a causative relationship between endogenous glucose storage in the form of glycogen, resistance to oxidative stress and organismal aging in *Caenorhabditis elegans.* We find that glycogen accumulated on high dietary glucose limits *C. elegans* longevity. Glucose released from glycogen and used for NADPH/glutathione reduction renders nematodes and human hepatocytes more resistant against oxidative stress. Exposure to low levels of oxidants or genetic inhibition of glycogen synthase depletes glycogen stores and extends the lifespan of animals fed a high glucose diet in an AMPK-dependent manner. Moreover, glycogen interferes with low insulin signalling and accelerates aging of long-lived *daf-2* worms fed a high glucose diet. Considering its extensive evolutionary conservation, our results suggest that glycogen metabolism might also have a role in mammalian aging.

## Abstract (web summary)

Glycogen is a storage form of glucose in cells. Here, Gusarov *et al*. show that glycogen-derived glucose can be used to quickly regenerate the antioxidant glutathione and that inhibiting glycogen synthesis extends *C. elegans* lifespan, whereas glycogen accumulation drives organismal ageing in worms.

All organisms employ similar pathways of glucose utilization and storage. Dietary carbohydrates are converted to glucose, which eventually donates its carbons to most other synthesized biomolecules. Glucose also serves as a major energy source (ATP production) and fuel for recycling reducing equivalents (NADPH and glutathione (GSH)). Surplus glucose is stored as glycogen or triglycerides for future energy needs. However, sugar overconsumption is uniformly detrimental to animals, as it leads to excessive body weight^1^, early onset of many disorders^2^,^3^ and reduced lifespan^4^,^5^. In contrast, caloric restriction extends the lifespan of both worms and mammals^6^,^7^,^8^. Despite extensive research, the fundamental mechanisms underlying the detrimental effects of glucose have not been well established.

*Caenorhabditis elegans* is an excellent model organism in which to study the effects of glucose on animal physiology and aging^9^,^10^,^11^,^12^. It has been proposed that activation of the insulin/insulin-like growth factor-1 signaling (IIS) pathway is the cause of reduced lifespan in worms fed a glucose-rich diet^13^. While long-lived mutant (*daf-2*) with reduced IIS also exhibited a short lifespan if exposed to glucose^13^, arguing that it is sensitive to a high glucose diet. Recent studies implicated the increased lipid production in worms’ tolerance to a high glucose diet, indicating that the lowering of cellular glucose by converting it to lipids can be beneficial^9^. Also, accelerated degradation of the products of glucose catabolism renders worms resistant to high glucose^10^. Thus the activation of any glucose-utilization pathway appears to be beneficial for the lifespan^9^,^10^.

The elevated level of reactive oxygen species (ROS) has been associated with a high glucose diet, albeit only in very old animals^10^, suggesting that in younger animals that may not be the case. Chemical inhibition of glucose metabolism also increased ROS formation and extended the nematode lifespan^11^. The traditional view of ROS as damaging agents responsible for the aging process has gradually been revised^14^,^15^, and it is now widely accepted that a certain level of ROS is necessary for normal cellular function and actually promotes longevity^16^,^17^,^18^. Sublethal doses of oxidants, or elevated endogenous ROS, were shown to extend the *C. elegans* lifespan^18^,^19^,^20^,^21^. In contrast, antioxidants often fail to influence lifespan and may actually accelerate aging^19^,^21^,^22^. We used *C. elegans* to examine the mechanism and role of ROS in glucose-mediated life shortening.

Here we show that glycogen, accumulated on a high glucose diet, drives cellular thiol reduction and thereby protects *C. elegans* and human hepatocytes against oxidative stress. In contrast, high glycogen depot shortens worm’s lifespan. Knocking down glycogen synthase or using oxidants to reduce glycogen stores extends lifespan of *C. elegans* in AMP-activated protein kinase (AMPK)-dependent manner. The evolutionary conservation of glycogen metabolism suggests that the glycogen-mediated impact on cellular redox status and aging might be universal.

## Results

### Glucose renders C. elegans resistant to oxidative stress

**Figure 1:**
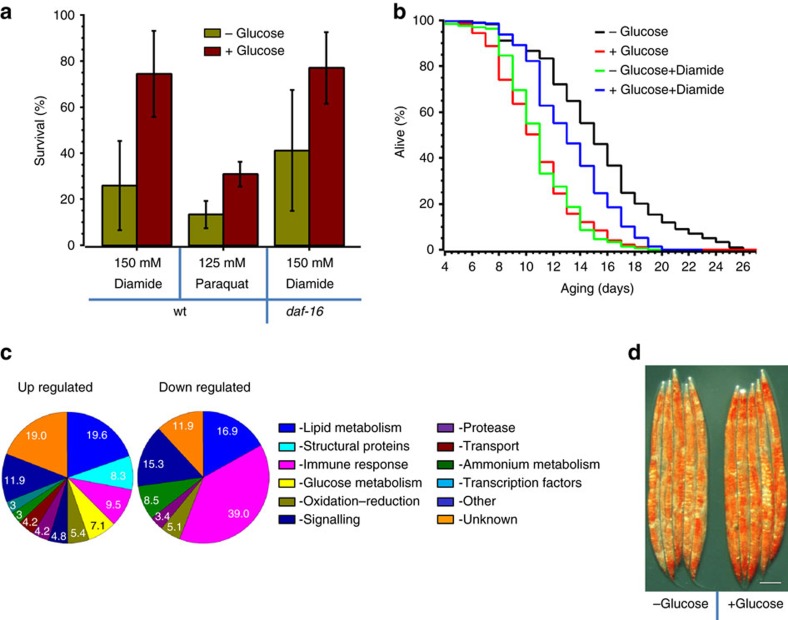
Dietary glucose protects *C. elegans* from oxidative stress. (**a**) Dietary glucose protects wt and *daf-16* worms against 125 mM paraquat (*n*=190, *P*=0.0006) or 150 mM diamide (wt: *n*=170 *P*=0.0057; *daf-16*: *n*=140, *P*=0.0284). L4 stage worms were placed on NGM plates with sorbitol (green) or glucose (red) for ∼20 h at 20 °C and then treated with the indicated oxidants. To compensate for glucose-induced osmolarity, sorbitol (a non-digestible sugar) was added to the diet of a control group. Graph shows the average survival±s.d. from three independent experiments. Approximately 50 worms were used per condition per each experiment. *P* values were calculated with respect to control animals in the same experiment using Student's *t*-Test. See Methods section for details. (**b**) Glucose in the diet protects against chronic exposure to high level of diamide (15 mM). The average curve from three independent experiments is plotted (see also Table 1 and Supplementary Table 2). (**c**) Transcriptional response to dietary glucose in *C. elegans.* Pie diagrams demonstrating major functional classes of genes upregulated (left panel) and downregulated (right panel) by glucose diet. L4 stage worms were placed on NGM plates with or without glucose for ∼20 h at 20 °C. Numbers indicate the percentage of genes of the given class. Full list of differentially expressed genes is presented in Supplementary Table 1. (**d**) Lipids accumulation in response to a high glucose diet. L4 stage wt worms were placed on NGM plates+/−glucose for 3 days at 20 °C followed by staining with Oil Red O. Scale bar 0.15 mm.

Typically, resistance to oxidative stress correlates with longevity^23^. For example, long-lived *daf-2* are more resistant to oxidants than are wild-type (wt) worms, and short-lived *daf-16* are sensitive^24^,^25^,^26^. Surprisingly, however, we found that a high glucose diet, which substantially shortens the *C. elegans* lifespan (Fig. 1b)^10^,^11^,^13^, also greatly protects worms against oxidants, such as diamide and paraquat (Fig. 1a). This observations seems even more surprising, as earlier microarray studies did not detect induction of any known oxidative stress genes in response to glucose^13^. This is, however, supported by another observation of glucose-induced resistance to a different oxidant, juglone^27^. Moreover, expression of the oxidative stress marker gene, *sod-3::GFP*, was suppressed by glucose^13^. Many cytoprotective genes including *sod-3* are regulated by the IIS-dependent transcription factor DAF-16. Because glucose also protects *daf-16* knockdown worms against diamide (Fig. 1a), we conclude that the antioxidant effect of glucose is not mediated by *daf-16*.

We next examined whether glucose also protected *C. elegans* against chronic exposure to an oxidant. As expected, a high level of diamide shortened the lifespan, whereas glucose abrogated this effect (Fig. 1b, Table 1). Taken together, these results demonstrate that glucose helps the animals to cope with oxidative stress.

### Glucose does not induce oxidative stress response in worms

It has been proposed that glucose consumption increases ROS in very old worms^10^. Hypothetically, elevated ROS on a high glucose diet may induce detoxifying genes, which would render animals more resistant to oxidative stress. To investigate this, we monitored the changes in global gene expression in response to glucose. RNA-seq differential expression analysis revealed 168 and 58 upregulated and downregulated genes, respectively, after 1 day of glucose feeding (Fig. 1c, Supplementary Table 1). As expected, glucose induced significant metabolic changes (Fig. 1c). About 20% of differentially regulated genes are involved in lipid metabolism. Amino acid and polyamine degradation genes are suppressed, whereas glutamine, asparagine, S-adenosylmethionine and glycerol-3-phosphate biosynthesis are upregulated. These results indicate that sugar consumption promotes net amino acid biosynthesis, sphingolipid and phospholipid metabolism and lipid storage in the form of triglycerides (Supplementary Table 1). Indeed, in agreement with previously published data, glucose-fed worms accumulated more lipids compared to worms on regular diet (Fig. 1d)^9^,^11^,^28^,^29^. As the elevated lipid storage is a hallmark of sugar overconsumption in mammals, *C. elegans* can be viewed as a relevant proxy model to study the mechanism of glucose toxicity. Interestingly, the majority of downregulated genes are related to immune response (Supplementary Table 1). However, since glucose shortens the lifespan of *C. elegans* on either live or killed *Escherichia coli* or on non-pathogenic *B. subtilis*, its toxicity cannot be attributed to enhanced pathogenicity^13^,^30^.

Notably, the list of genes upregulated by glucose is not enriched with ROS-detoxifying or ROS-damage repair genes (Supplementary Table 1). Out of the 115 genes known to be involved in the oxidative stress response (Worm Base gene ontology term GO:0006979), only one hypothetical thioredoxine-like gene (*W01B11.6*) was modestly induced by glucose. Thus glucose-mediated ROS resistance (Fig. 1a) cannot be explained by hormesis and upregulation of protective genes, but instead it appears to activate a pre-existent defense mechanism.

### Glucose-mediated GSH reduction protects against oxidants

**Figure 2:**
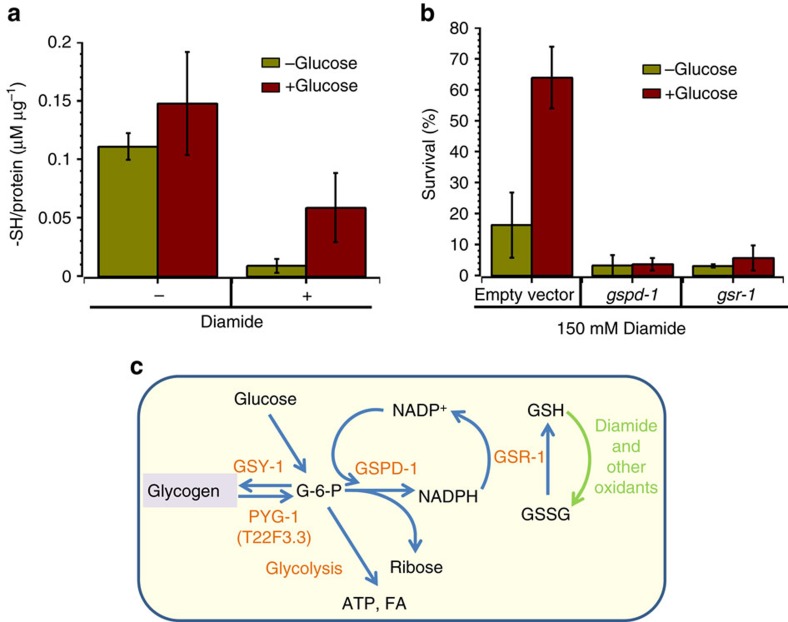
Glucose-driven glutathione reduction is the major defense against oxidants. (**a**) Glucose promotes cellular thiols reduction. L4 worms were placed on NGM plates with or without glucose. Two-day-old adults were picked and incubated for 10 min with (*n*=400, *P*=0.035) or without (*n*=400, *P*=0.13) non-lethal concentration of diamide. Total cellular reduced thiols were quantified in worm lysates by reaction with DTNB (see Methods section) Graph shows the mean±s.e. from four independent experiments. (**b**) Endogenous glutathione is the major defense against oxidants. Glutathione reductase (*gsr-1*) or glucose-6-phosphat dehydrogenase (*gspd-1*) were knocked down by RNAi, followed by diamide treatment as in Fig. 1a. Graph shows the mean±s.d. from at least three independent experiments (e.v.: *n*=140, *P*=0.0003; *gsr-1*: *n*=120, *P*=0.129; *gspd-1*: *n*=110, *P*=0.434). *P* values were calculated with respect to control animals in the same experiment using Student's t-Test. See also Supplementary Fig. 1. (**c**) Major pathways of glucose utilization and storage. Upon entering the cell, glucose is phosphorylated and is utilized for ATP production and the biosynthesis of macromolecules. Excess glucose is stored as glycogen or converted to fatty acids (FA). A large portion of glucose is used for NADP^+^ reduction, which is required for subsequent reduction of oxidized glutathione and thioredoxin. Glucose thus acts as a powerful (albeit indirect) antioxidant. GSY-1, glycogen synthase; PYG-1(T22F3.3), glycogen phosphorylase; GSPD-1, glucose-6-phosphate dehydrogenase; GSR-1, glutathione reductase.

With the exception of ROS-neutralizing enzymes, GSH acts as the major cellular defense against oxidants^31^. For example, after oxidation to disulfide (GSSG), it is re-reduced to GSH by glutathione reductase (GSR), which uses NADPH as the electron donor^32^. The pentose phosphate pathway (PPP) generates most of the cellular NADPH from glucose-6-P (Fig. 2c). Thus surplus glucose should accelerate GSSG reduction and increase oxidant scavenging. Indeed, we detected an increase of cellular reduced thiols in worms fed a high glucose diet (Fig. 2a). Moreover, the glucose-fed worms maintain their level of reduced thiols in response to diamide much better than worms on a regular diet (Fig. 2a). To further implicate PPP in glucose-mediated resistance to oxidants, we used RNAi to knockdown glucose-6-P dehydrogenase (*gspd-1*) or GSR (*gsr-1*) (Fig. 2c). Worms lacking either of these enzymes became more sensitive to diamide and displayed much weaker glucose-mediated protection against oxidants (Fig. 2b and Supplementary Fig. 1). These results implicate the enhanced cellular ability to reduce NADP^+^ and GSSG in glucose-mediated protection against oxidative stress and imply that GSH is the major defense system against acute oxidative stress.

### Glycogen is an evolutionary conserved antioxidant

**Figure 3:**
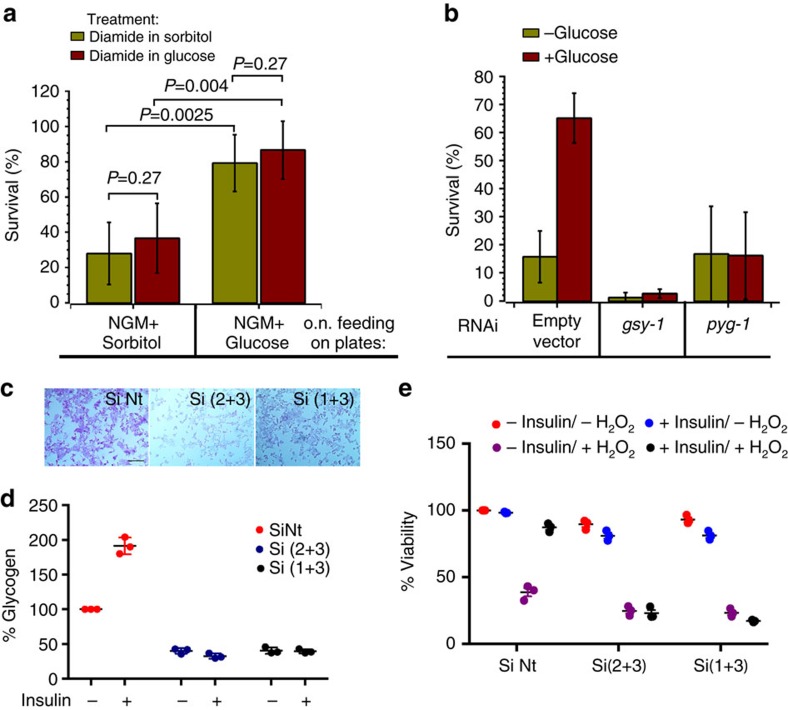
Glycogen protects *C. elegans* and hepatocytes against oxidative stress. All graphs show the average survival±s.d. from at least three independent experiments. Approximately 50 worms were used per condition per each experiment. (**a**) Stored glucose metabolite(s), not glucose in the media, protects *C. elegans* from oxidants. L4 stage animals grown on NGM plates+sorbitol or +glucose for 20 h were picked and transferred to M9 containing sorbitol and diamide (green bars) or glucose and diamide (red bars). (**b**) Glycogen storage is required for *C. elegans* protection against oxidants. N2 worms fed on NGM plates seeded with *E. coli* HT115 harbouring either empty plasmid vector or plasmid vectors expressing *gsy-1* or *pyg-1* RNAi. L4 stage animals were transferred to NGM plates with sorbitol (green bars) or with glucose (red bars) for 20 h and then treated with 150 mM diamide (e.v.: *n*=130, *P*=0.00001; *gsy-1*: *n*=155, *P*=0.105; *pyg-1*: *n*=180, *P*=0.479). *P* values were calculated with respect to control animals in the same experiment using Student's t-Test. See also Supplementary Fig. 2. (**c**) PAS staining of HepG2 cells after glycogen synthase 2 knockdown by siRNA. SiNt, non target siRNA; Si(2+3), combination of siRNA GYS2.2 and GYS2.3; Si(1+3), combination of siRNA GYS2.1 and GYS2.3. Scale bar 100 μm. (**d**) Relative glycogen content in HepG2 cells treated with insulin and GYS-2 siRNA. Error bars represent s.d. of three independent experiments. (**e**) Glycogen protects hepatocytes against peroxide. HepG2 cell were depleted of glycogen synthase 2 by siRNAi and then incubated with 50 μM H_2_O_2_ for 30 min. Cell viability was assayed after 24 h. Insulin was added to a final concentration of 25 μM. Error bars represent s.d. of three independent experiments.

Because the *de novo* synthesis of cytoprotective enzymes is not necessary for glucose-mediated resistance to oxidants (Supplementary Table 1), we expected that the addition of glucose while challenging animals with an oxidant should be sufficient to protect them. Strikingly, however, the same concentration of glucose given to the worms during the oxidant treatment failed to improve survival (Fig. 3a), suggesting that a product of glucose metabolism, and not dietary glucose itself, is protective against oxidants.

In most organisms, excess glucose is promptly stored as glycogen (Fig. 2c). Glycogen synthesis/phosphorolysis is regulated via phosphorylation of cognate enzymes, absent a requirement for gene activation or *de novo* protein synthesis. Therefore, glucose can be rapidly retrieved from glycogen. RNAi depletion of glycogen synthase (*gsy-1*) abolished glycogen accumulation, regardless of diet (see below). In the absence of GSY-1, *C. elegans* become very sensitive to diamide and dietary glucose failed to efficiently protect worms against oxidative stress (Fig. 3b and Supplementary Fig. 2). Consistently, glycogen phosphorylase depletion (*pyg-1*, *T22F3.3*) increased glycogen accumulation (see below) but failed to protect worms against oxidative stress (Fig. 3b and Supplementary Fig. 2). Thus increased glycogen provides the driving force for the rapid reduction of NADP^+^ and GSSG and detoxification of oxidants in nematodes. Although lipids are also stored on a high glucose diet (Fig. 1d)^9^,^11^,^28^,^29^, their breakdown is much slower than glycogen phosphorolysis and NADH and FADH_2_ generated as a result of β-oxidation cannot support GSSG reduction^33^.

To determine whether the glycogen-dependent protection against oxidative stress also occurs in mammalian cells, we depleted glycogen synthase (*GYS-2*) in human hepatocytes (Fig. 3c and Supplementary Fig. 3). Liver cells incubated with and without insulin correspond to a high and low glucose dietary state, respectively. Indeed, the high level of glycogen accumulated only in the presence of insulin (Fig. 3d) and that efficiently protected hepatocytes against hydrogen peroxide toxicity (Fig. 3e). Depletion of glycogen synthase prevented glycogen accumulation and sensitized hepatocytes to ROS (Fig. 3d,e). Together, these results demonstrate that the phenomenon of glycogen-mediated protection against oxidative stress is evolutionary conserved from worms to humans.

### Glycogen limits C. elegans lifespan on a high glucose diet

Mild oxidative stress delays *C. elegans* aging, supposedly due to the hormesis effect, that is, mobilization of cellular defense systems that would otherwise remain silent^19^,^21^. Therefore, decreased lifespan in response to dietary glucose could be explained by the glycogen-driven ability to prevent protein thiol oxidation. This would result in interference with redox-sensitive thiol signalling and downregulation of cytoprotective gene transcription.

**Figure 4:**
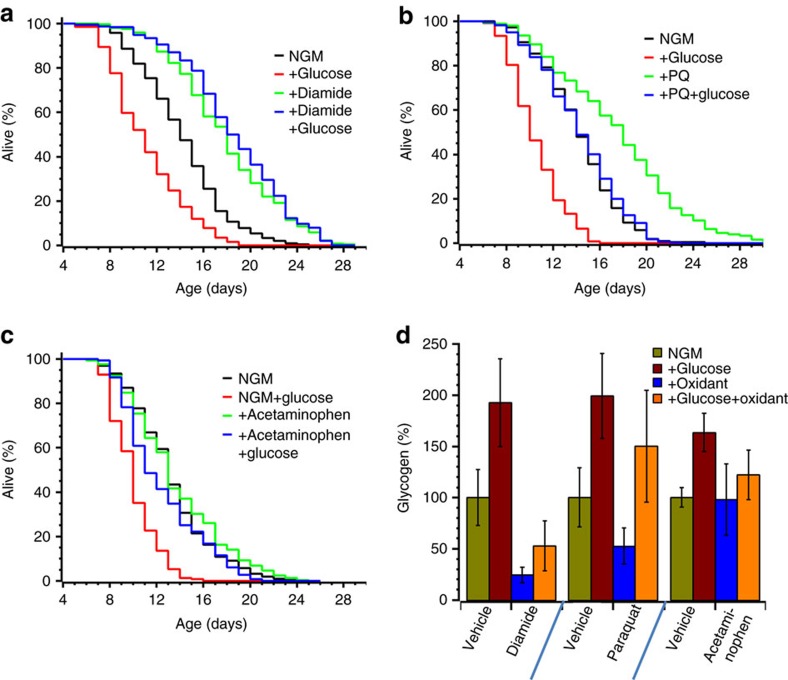
Glycogen depletion by oxidants delays aging. (**a**–**c**) The short lifespan of glucose-fed *C. elegans* is reversed by 5 mM diamide (**a**), 0.2 mM paraquat (**b**) and 5 mM acetaminophen (**c**). Half of L4 staged animals were placed on NGM plates +/−glucose (red and black curves), the other half was transferred to NGM plates+/−glucose supplemented with diamide (**a**), paraquat (**b**) or acetaminophen (**c**). For each graph, the average curve from three independent experiments is plotted (see also Table 1 and Supplementary Table 2). (**d**) Glycogen content is increased in worms fed a high glucose diet and is depleted by oxidants. Worms were grown and fed as in panel **a**–**c**. Glycogen content of 3-day-old animals was determined by the enzymatic assay and normalized to total protein. The graph shows changes in glycogen levels from at least three independent experiments (%±s.d.) between the experimental and control groups of animals (see also Supplementary Table 3).

To test this hypothesis, we first examined whether lifespan shortening in glucose-fed worms could be reversed by low level of exogenous oxidants. Indeed, diamide eliminated the negative effect of glucose on *C. elegans* lifespan (Fig. 4a, Table 1). Paraquat also decreased glucose toxicity although its effect was smaller than that of diamide, perhaps because paraquat utilizes some of NADPH, which is required for GSSG reduction, to generate ROS^34^ (Fig. 4b, Table 1). Furthermore, ubiquitous anti-inflammatory drug acetaminophen (Tylenol) protected worms against glucose toxicity (Fig. 4c). The major side effect of acetaminophen in animals is liver damage caused by severe oxidative stress, which accounts for many life-threatening cases of drug overdose every year^35^,^36^. Acetaminophen conjugates with cellular GSH, depleting hepatocytes of their primary antioxidant^35^,^36^. Note that, similar to paraquat or diamide, a very low concentration of acetaminophen also extends *C. elegans* lifespan on a regular diet^37^. Together our results demonstrate that oxidants and glucose cancel out each other’s negative effect on the lifespan (Figs 1b and 4).

**Figure 5:**
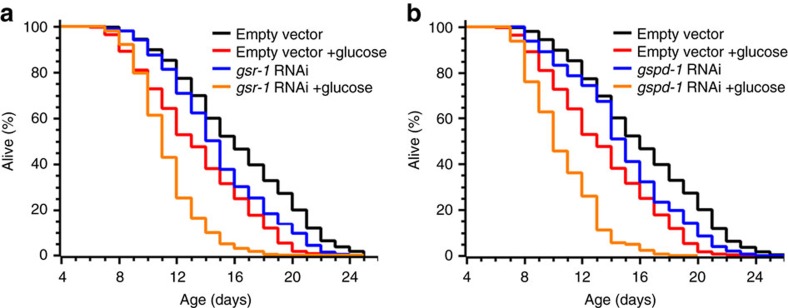
Blocking the endogenous antioxidant system does not influence glucose-mediated life shortening. (**a** and **b**) Wt worms grew on NGM plates seeded with *E. coli* HT115 harbouring either an empty vector (black and red curves) or a plasmid expressing either double-stranded *gsr-1* (**a**) or *gspd-1* (**b**) RNAi (blue and orange curves). L4 stage animals were transferred to NGM plates without (black and blue curves) or with (red and orange curves) glucose (see also Supplementary Table 2). For RNAi efficiency, see Fig. 2b.

In principle, either low glycogen or elevated ROS could initiate a signal leading to extended lifespan. To discriminate between these two possibilities, we first diminished the reduction of thiols by knocking down GSR (*gsr-1*) or glucose-6-phosphate dehydrogenase (*gspd-1*), the enzyme which generates NADPH, the cofactor required by all GSSG-reducing enzymes (Fig. 2c). GSR or GSPD depletion compromises scavenging of endogenous, as well as exogenously added, ROS without a significant effect on glycogen stores (Supplementary Fig. 4a)^32^. However, both GSR- and GSPD-deficient animals did not live longer than wt and remained susceptible to glucose-mediated life shortening (Fig. 5, Table 1). Thus the low level of reduced GSH, and the resulting elevated ROS, does not increase the lifespan. Instead, the oxidant-mediated depletion of glycogen stores appears to be the primary cause of life extension by oxidants.

To confirm that oxidants do, indeed, deplete glycogen, we monitored the glycogen level in worm cells lysates using an enzymatic assay or in live animals by iodine staining (Fig. 4d and Supplementary Fig. 4b). Glucose-fed worms accumulated twofold more glycogen than worms fed a regular diet (Fig. 4d, Supplementary Table 3). Challenging worms with diamide, paraquat or acetaminophen (under the same conditions that resulted in life extension) depleted the glycogen stores (Fig. 4d, Supplementary Fig. 4b and Supplementary Table 3). Thus glycogen is depleted in response to oxidative stress, and notably, the magnitude of lifespan extension by oxidants or shortening by glucose is evidently correlated with changes in glycogen content (Fig. 4 and Supplementary Fig. 4c).

**Figure 6:**
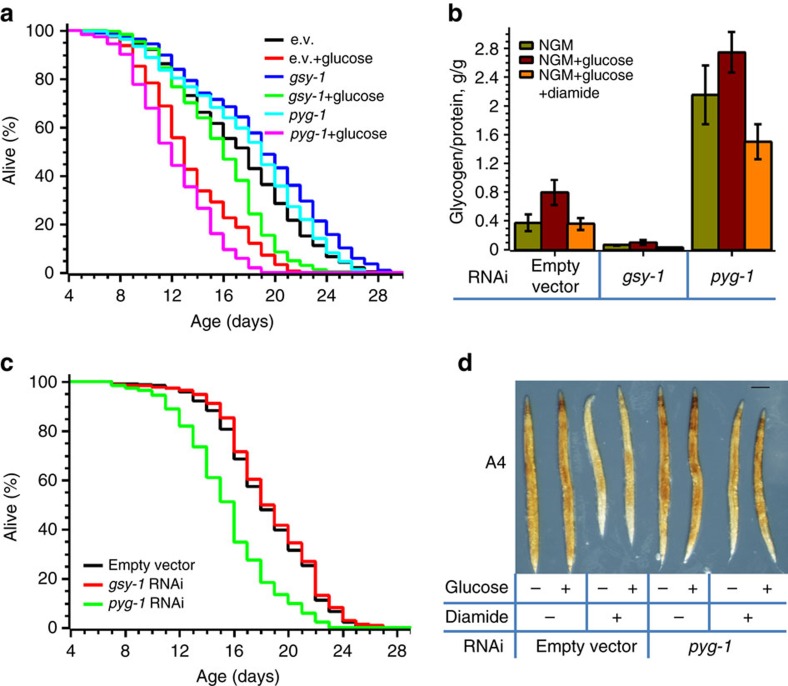
Glycogen accumulation is responsible for glucose-mediated life shortening. (**a**) Worms lacking glycogen deposition live much longer on a high glucose diet. N2 worms were fed on NGM plates seeded with *E. coli* HT115 harbouring either empty vector (black and red curves) or plasmids expressing either *gsy-1* (blue and green curves) or *pyg-1* (cyan and magenta curves) RNAi. L4 stage animals were transferred to NGM plates without (black, blue and cyan curves) or with (red, green and magenta curves) glucose. The graph shows the average of three independent experiments (see also Supplementary Table 2). (**b**) Glycogen content in *gsy-1* and *pyg-1* knockdown worms. Animals were grown on NGM (green bars), NGM+2% glucose (red bars) or NGM+2% glucose+5 mM diamide (orange bars) agar plates as in panel **a** or **c**. Seven-day-old worms were then picked, washed and the amount of glycogen determined and normalized to the total protein. The graph shows the average±s.d. of at least three independent experiments (see also Supplementary Table 3). (**c**) Diamide fails to extend the short lifespan of glycogen phosphorylase-deficient worms fed on high glucose diet. N2 worms were fed on NGM plates seeded with *E. coli* HT115 harbouring either empty vector (black curve) or plasmid vector expressing either *gsy-1* (red curve) or *pyg-1* (green curve) RNAi. L4 stage animals were transferred to NGM plates with 2% glucose and 5 mM diamide. The graph shows the average of three independent experiments (see also Supplementary Table 2). (**d**) Diamide does not efficiently deplete glycogen in *pyg-1*-deficient worms. A representative image of four-day-old worms stained for glycogen with iodine is shown. Scale bar 0.15 mm. Experimental conditions were as in panel **c**.

To prove that glycogen diminishes the *C. elegans* lifespan, we knocked down glycogen synthase (*gsy-1*) or glycogen phosphorylase (*pyg-1*, *T22F3.3*), which breaks down glycogen (Fig. 2c). Irrespective of diet, *gsy-1* depletion prevented glycogen accumulation (Fig. 6b,d, Supplementary Table 3) and increased the lifespan of glucose-fed worms (Fig. 6a, Table 1). Curiously, *gsy-1* depletion only through the development phase and first 4 days of adulthood was as effective against glucose-mediated toxicity as its life-long depletion (Supplementary Fig. 5). This result indicates that glycogen accumulation in early age is more detrimental to animal health.

As expected, the deficiency of glycogen phosphorylase increased the amount of stored glycogen resulting in even shorter lifespan on a high glucose diet (Fig. 6a,b, Table 1). However, on glucose-free media, *pyg-1*-deficient worms, while still accumulating glycogen, did not live shorter (Fig. 6a,b), indicating that glycogen accelerates aging only on a high glucose diet.

To demonstrate that the oxidants are not capable of reversing the negative effect of glucose on lifespan, if the glycogen level remains high, we fed wt, *gsy-1* and *pyg-1* worms both glucose and diamide. As expected, diamide depleted glycogen in wt and *gsy-1* animals (Fig. 6b,d) and increased their lifespan (Fig. 6c and Table 1). In contrast, diamide failed to increase the lifespan of *pyg-1*-deficient animals, which accumulated the high level of glycogen (Fig. 6b,c).

Together these results demonstrate that it is not the antioxidant capacity (high GSH/GSSG ratio) or low ROS but rather endogenous glycogen that signals to reduce the lifespan of *C. elegans* fed a high glucose diet. Diamide and other oxidants deplete glycogen via the PPP/GSH pathway, thereby switching off this life-shortening signal (Fig. 2c).

### Glycogen interferes with the daf-2 antiaging signalling

Inhibition of IIS substantially extends the *C. elegans* lifespan^38^. Mutations in the insulin/insulin-like growth factor receptor gene, *daf-2*, induce *daf-16-*mediated transcription of many cytoprotective and antiaging genes^39^,^40^,^41^. Dietary glucose is thought to activate *DAF-2* to repress *daf-16*-regulated transcription^13^. DAF-16 enters the nucleus in glucose-fed *daf-2* worms, but the antiaging properties of these animals are somehow subjugated by glucose^13^.

**Figure 7:**
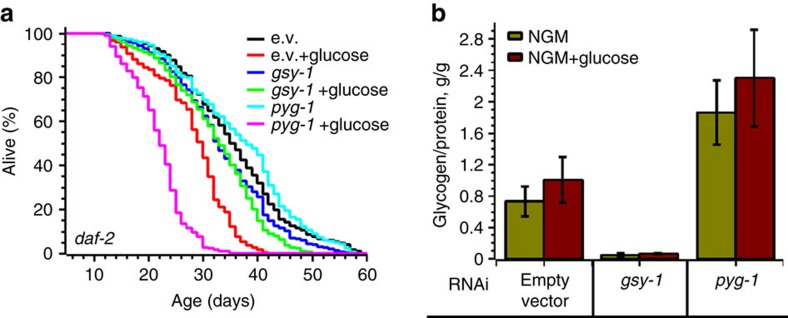
Glycogen is responsible for glucose-mediated life shortening of *daf-2* worms. (**a**) Inverse correlation between glycogen production and lifespan of *daf-2* (e1370, hypomorph) worms fed a high glucose diet. Worms were grown on NGM plates seeded with *E. coli* HT115 harbouring either empty vector (black and red curves) or plasmids expressing either *gsy-1* (blue and green curves) or *pyg-1* (cyan and magenta curves) RNAi. L4 stage animals were then transferred on NGM plates without (black, blue and cyan curves) or with (red, green and magenta curves) glucose. The graph shows the average of three independent experiments (see also Supplementary Table 2). (**b**) Glycogen content in *daf-2* worms depleted of *gsy-1* or *pyg-1*. Worms were grown as in panel **a** on NGM (green bars) or NGM+2% glucose (red bars) plates. Seven-day-old worms were picked, washed and the amount of glycogen determined and normalized to the total protein. The graph shows the average±s.d. of at least three independent experiments (see also Supplementary Table 3).

Worms, exposed to dietary glucose postdevelopmentally, increased glycogen accumulation by ∼30% and live ∼5 days shorter than *daf-2* control animals fed a regular diet (Fig. 7a,b, Supplementary Fig. 6, Supplementary Table 3). To determine whether glycogen initiates a signal that decreased longevity of glucose fed *daf-2* worms, we used RNAi to deplete *gsy-1* (Fig. 7b and Supplementary Fig. 6). Although *gsy-1 daf-2* animals, which do not make glycogen, live ∼5% shorter compared to *daf-2* worms, their lifespan was not further affected by glucose (Fig. 7a and Table 2). Consistently, *pyg-1* RNAi further increase glycogen level in *daf-2* worms and dramatically shorten their lifespan on a high glucose diet (Fig. 7a and Table 2), as if more glycogen is required to counterbalance low IIS.

Note that on a glucose-free diet the glycogen level is higher in both *daf-2* and *pyg-1 daf-2* worms compared to wt (Figs 6b and 7b). However, those animals do not live shorter. These results demonstrate again that glucose alone (such as in the case of *gsy-1* worms), or glycogen alone, do not shorten the lifespan. Glycogen accumulated in the presence of high glucose interferes with low IIS, resulting in a shortened lifespan of *daf-2* animals.

### Glycogen regulates SOD-3 and aging independently of DAF-16

FOXO/DAF-16 transcription factor contributes greatly to *C. elegans* longevity^41^. Expression of *sod-3* in intestinal cells is a reliable marker of DAF-16 antiaging activity^42^. In accordance with previously published data^13^, we found that a high glucose diet decreased *sod-3* expression by ∼50% in wt and by ∼30% in *daf-2* worms (Supplementary Fig. 7 and Supplementary Table 4). Depletion of glycogen synthase (*gsy-1*) increased *sod-3* expression ∼2.5 times in wt and ∼1.5 times in *daf-2* worms (Supplementary Fig. 7). Remarkably, glucose fails to inhibit *sod-3::GFP* accumulation in glycogen-depleted worms (Supplementary Fig. 7). We noticed that effect of *daf-2* mutation on *sod-3* expression is much stronger than from glycogen depletion and results in GFP accumulation mainly in the head area (compare Supplementary Fig. 7a and 7c). In contrast, glycogen depletion mostly increases fluorescence in the tail area (Supplementary Fig. 7). To clarify this, we subjected wt worms to *gsy-1* and *daf-16* double RNAi treatment. Surprisingly, the depletion of DAF-16 increased the tail accumulation of GFP in wt worms and had no effect on *sod-3* expression in *gsy-1*-deficient worms (Supplementary Fig. 7e). Together these results indicate that the tail expression of *sod-3* is not a marker of DAF-16-mediated transcription.

**Figure 8:**
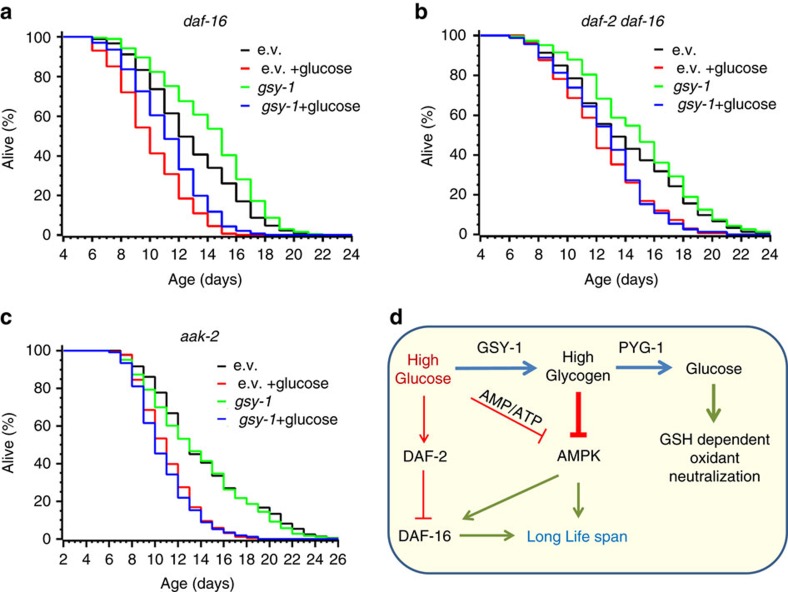
AMPK is required for life extension in glycogen-depleted *C. elegans*. (**a**–**c**) Depletion of glycogen extends the lifespan of *daf-16* (**a**) and *daf-2 daf-16* (**b**) worms but fails to increase the longevity of *aak-2* animals (**c**). Worms were fed on NGM plates seeded with *E. coli* HT115 harbouring either empty vector (black and red curves) or plasmids expressing *gsy-1* (blue and green curves) RNAi. L4 animals were transferred to NGM plates without (black and green curves) or with (red and blue curves) glucose. The graph shows the average of three independent experiments (see also Table 2 and Supplementary Table 2). (**d**) Interplay between glycogen and insulin-like signalling controls *C. elegans* lifespan. A high glucose diet simultaneously activates insulin-like signalling and induces glycogen storage. IIS suppression of DAF-16-mediated transcription and inhibition of AMPK by glycogen shorten the lifespan. Pro-aging glycogen signalling overcomes the antiaging effect of IIS inhibition (*daf-2*). Inactivation of glycogen synthesis (*gsy-1*) prevents glycogen accumulation, relieves AMPK and extends the lifespan in wt, *daf-16* and *daf-2* worms on a high glucose diet. Oxidants force the glucose flow through PPP to regenerate cellular GSH, thus depleting glycogen stores and extending the lifespan.

To further address the role of *daf-16* in glycogen-mediated life shortening, we depleted *gsy-1* in *daf-16* and *daf-2 daf-16* worms. In our experimental set-up (adults only glucose treatment), glucose decreased the lifespan of *daf-16* and, to a lesser extent, of *daf-2 daf-16* animals (Fig. 8a,b, Supplementary Table 2). Remarkably, *gsy-1* RNAi increased the lifespan of both these strains irrespective of their diet. These results indicate that glycogen shortens the lifespan independently of *daf-16* (Fig. 8 and Table 2).

### AMPK is required for high glucose tolerance in *gsy-1* worms

Glycogen dramatically shortens the lifespan of *daf-2* animals on a high glucose diet (Fig. 7a), implying that it must interfere with a certain DAF-2-regulated antiaging pathway. DAF-16 and AMPK are both required for the full lifespan extension of *daf-2* animals^43^,^44^. Moreover, the deletion of *aak-2*, an AMPK ortholog in *C. elegans*, shortens the lifespan of *daf-2* and *daf-16* (refs 43, 45, 46). Paraquat treatment does not extend *aak-2* lifespan^47^. Finally, in human cells, glycogen directly binds and sequesters AMPK to downregulate its activity^48^. Together, these observations suggest that AMPK is a target of glycogen-induced regulation of *C. elegans* lifespan. To address this possibility, we depleted gsy-1 in *aak-2* worms and compared their lifespan on regular and high glucose diet. In agreement with the published data, glucose further shortened the lifespan of *aak-2* (Fig. 8c)^13^. However, gsy-1 depletion did not extend its lifespan on a high glucose diet (Fig. 8c and Table 2), indicating that AMPK is indeed required for life extension in the absence of glycogen. Thus the lifespan of *C. elegans* is determined by a combination of *daf-2-* and glycogen-to-AMPK-mediated signalling (Fig. 8d).

## Discussion

Glucose is essential for animal life. Yet, its excess has been linked to a multitude of pathological conditions^2^,^3^ and diminished lifespan across evolutionary genera^4^,^5^,^13^. Several attempts have been made to explain glucose toxicity, and the IIS pathway was proposed to be the major target^13^. However, genetic inactivation of IIS in worms does not mitigate the negative effect of glucose on lifespan^13^ (Fig. 7a), indicating that other pathways must be involved. Here we show that glycogen signalling plays the principal role in limiting the lifespan of nematodes fed a high glucose diet (Fig. 8d).

Our results indicate that glycogen and IIS are the two distinct signalling pathways controlling the lifespan in nematodes (Fig. 8d). On a high glucose diet, IIS is activated to suppress DAF-16-dependent transcription. Simultaneously, accumulation of glycogen inhibits AMPK activity. Together this leads to a dramatic shortening of lifespan (Fig. 6a). Inhibition of glycogen synthesis by *gsy-1* RNAi relieves AMPK inhibition and, consistently, increases the lifespan of wt, *daf-2* and *daf-16* worms fed a high glucose diet (Figs 6a, 7a and 8). The latter indicates that glycogen and *daf-16* are parallel signalling pathways independently controlling the lifespan. In GSY-1-deficient worms fed glucose, DAF-2 signalling could still suppress DAF-16; however, this effect is countered by low glycogen, which relieves the AMPK inhibition (Fig. 8d), thereby promoting *daf-16* activation^49^.

Activation IIS by glucose explains why *gsy-1* worms on high glucose live slightly shorter comparing to wt on a regular diet (Fig. 6a). Conversely, in *daf-2* background, where the negative effect of glucose on lifespan via the IIS pathway is abrogated, *gsy-1* depletion completely restored the lifespan (Fig. 7a). Although inconsistent with published data^13^,^27^, the negative effect of glucose on the lifespan of *daf-16* worms, which we observe here, fits our model. Glycogen accumulated in *daf-16* worms shortens their lifespan via the AMPK pathway, while activation of IIS signalling by glucose decreases the lifespan of AMPK-deficient worms (Fig. 8).

Glycogen phosphorylase-deficient worms accumulate excess of glycogen and, in accordance with our model, live shorter on a high glucose diet, irrespective of IIS (Fig. 8d). However, on a glucose-free diet accumulation of glycogen does not compromise their lifespan (Figs 6a and 7a). This clearly demonstrates that both dietary glucose and glycogen accumulation are required for shortening the lifespan. AMPK is primarily regulated by AMP and ATP^43^,^50^ and its activity is oppositely controlled by AMP and glycogen in mammals^48^. Consistently, we found that the level of ATP is lower in *pyg-1*-deficient worms compared to wt (Supplementary Fig. 8), apparently, because most of cellular glucose is trapped in glycogen on sugar-free diet. Low ATP should oppose glycogen-dependent inhibition of AMPK (Fig. 8d).

Although the molecular details of glycogen-mediated regulation of AMPK remain to be characterized, it is reasonable to propose that the pharmacological inhibition of this pathway could be beneficial. Liver glycogen helps to maintain the steady level of circulating glucose between meals. However, the excess glycogen observed in many disorders^51^,^52^, including several types of glycogen-storage diseases and diabetes may be a root cause of concomitant pathologies. Patients with type I, VI and IX glycogen storage diseases accumulate high levels of glycogen and are at increased risk of developing cirrhosis, hepatocellular adenomas and carcinomas^51^,^52^. The role of glycogen in development of these pathologies, however, has never been directly addressed. Chronic inhibition of glycogen phosphorylase in rats causes liver inflammation and damage, supporting the link between glycogen excess and disease^53^. In contrast, adults with liver glycogen synthase deficiency are typically asymptomatic and rely on gluconeogenesis to meet their glucose needs^54^,^55^. Thus it is possible that liver glycogen is expendable during adulthood, and excess glycogen resulting from a high carbohydrate diet may actually be harmful.

Our experiments with *C. elegans* support this idea. *Gsy-1* animals produce very little glycogen. They manifest no abnormalities in adulthood, live ∼15% longer than wt animals and are resistant to a high glucose diet (Figs 6a and 7a). In contrast, worms that accumulate glycogen when fed a high sugar diet have a shortened lifespan, irrespective of their level of insulin signalling (Figs 6a and 7a). Thus one of the important outcomes of this study is that dietary glucose is not harmful unless it can be converted to glycogen.

Our findings offer the enticing possibility that the harm imposed by dietary glucose in adults could be reduced by suppressing liver glycogen accumulation with glycogen synthase inhibitors, or, alternatively, by promoting glycogen depletion with oxidants (Figs 2c and 4). A growing body of evidence associates mild oxidative stress, for example, from physical exercise^56^, with longer healthspan, while antioxidants have the opposite effect^22^. Our results indicate that glucose can be viewed as a ‘super’ antioxidant. Large glycogen depots allow much faster reduction of GSSG and therefore rapid ROS scavenging upon sudden oxidative insult (Figs 1a, 2a and 3a,e). Hence, by applying small amount of oxidants one can drain glycogen stores through PPP metabolism, relieve AMPK inhibition and restore normal lifespan of animals on a high glucose diet (Fig. 8). Considering the high evolutionary conservation of glycogen metabolism, we propose that limited amounts of a therapeutically accepted oxidant could, in principle, be used to lower hepatic glycogen stores.

## Methods

### Strains and growth conditions

Wt *C. elegans* (N2), CF1038 (*daf-16(mu86) I*), TG38 (aak-2(gt33)), CF1553 (*muIs84* ((pAD76) *sod-3p*::GFP+*rol-6*)), CF1580 (*daf-2*(e1370) III; *muIs84* ((pAD76) *sod-3p*::GFP+*rol-6*)), HT1890 (*daf-16(mgDf50) I; daf-2(e1370) III*) and CB1370 (*daf-2(e1370) III*) strains were obtained from the *Caenorhaditis* Genetics Center. The worms were handled according to standard methods^57^. Strains were grown on nematode growth media (NGM) agar plates at 20 °C except for the *daf-2* strain, which was grown at 15 °C until L4 stage. *E. coli* OP50 bacteria were grown overnight in LB and 50 μl were spread on top of the agar plates. Plates were incubated for ∼20 h at 25 °C and then for at least 1 h at 20 °C before worms were transferred onto them.

For RNAi experiments, eggs were isolated by treating adult hermaphrodites with alkaline hypochlorite and allowed to develop and grow for two generation on specific RNAi-expressing bacterial strains before being used or lifespan and stress-resistance analysis. First generation was used only in case worms fed with bacteria expressing *gspd-1* RNAi, as the second generation does not develop. *E. coli* HT115 strains harbouring plasmid expressing double-strand RNAi against *C.elegans gsy-1*, *pyg-1, gsr-1* and *gspd-1* genes were purchased from the Thermo Scientific collection and single-colony isolates were purified and sequenced to prove the presence of the correct insert. Overnight cultures of *E. coli* HT115 bacteria harbouring RNAi-expressing plasmid or empty plasmid vector control (pL4440) were grown in LB with 100 m kg ml^−1^ of carbenicillin, concentrated 4 times and 50 μl were spread atop NGM agar plates supplemented with 100 μg ml^−1^ carbenicillin and 1 mM IPTG. Seeded plates were incubated for at least 1 h at 20 °C before worms were transferred onto them.

### Lifespan analysis

Lifespans were monitored at 20 °C as described previously^58^,^59^. All experiments were repeated at least three times and ∼100 worms were used for each experiment. In all cases, stage L4 worms were used at *t*=0 for lifespan analyses. Worms were judged to be dead when they ceased pharyngeal pumping and did not respond to prodding with a platinum wire. Worms with internal hatching were removed from the plates and not included in lifespan calculations. Data were analysed and Boltzmann sigmoid survival curves generated using the SciDAVis statistical analysis software package. Mean lifespans were compared in Microsoft Excel using the Student’s *t*-test, assuming one-tailed distribution and two-sample equal variance. All lifespan plots represent the composites of all independent experiments tabulated in Supplementary Table 2.

In all experiments, worms were allowed to develop until stage L4 on NGM plates at 20 °C (with the exception of *daf-2*, which developed at 15 °C) and then transferred to the plates with the indicated additives. Where indicated, glucose (2% final concentration) was mixed in NGM plates. Oxidants were evenly distributed on wet NGM (or NGM with glucose) plates to reach to a final concentration of: 5 mM diamide, 0.2 mM paraquat or 5 mM acetaminophen. Diamide and paraquate stock solutions were prepared in water, acetaminophen was dissolved in ethanol and the same concentration of the solvent was used in control plates. Plates were dried for 30 min before seeding with bacteria. As all oxidants have some bacteriostatic effect, the overnight bacterial cultures were concentrated 8 times and 50 μl were spread atop agar plates.

### Stress-resistance assays

Resistance to diamide or paraquat was assessed essentially as described^60^. Worms were allowed to develop and grow on agar plates at 20 °C until they reached stage L4 and then transferred to NGM+50 μg ml^−1^ kanamycin plates with 2% sorbitol or glucose and incubated for ∼20 h at 20 °C. It was important to add sorbitol in control to match the osmolarity of high glucose diet. Kanamycin prevents sorbitol and glucose consumption by bacteria. Worms (40–80 animals per each experimental condition) were collected into microcentrifuge tubes with 0.25 ml of M9 buffer with sorbitol or glucose supplemented with oxidant. The final concentration of diamide was 125 or 150 mM, and paraquat—125 mM. Tubes were incubated at 20 °C with agitation for 1–2 h. To remove the oxidant, worms were washed in M9 buffer three times and then transferred to NGM plates seeded with bacteria. Plates were incubated for 48 h at 20 °C and then live worms were counted and the survival rate was calculated as a ratio of those alive to the total number of worms transferred to the plate after oxidant treatment. All experiments were repeated at least three times and the average±s.d. presented in the Figs.

### Determination of glycogen content in *C. elegans*

Worms were allowed to develop and grow on agar plates at 20 °C until they reached stage L4 and then transferred to NGM plates with or without glucose. At certain ages, worms were collected in micro-centrifugal tubes, washed quickly two times with distilled water, most of the liquid was removed and the worms were flash frozen in liquid nitrogen and stored at −80 °C until further analysed. For analysis, worms were resuspended in ∼70 μl of dH_2_O and immediately boiled for 5 min to inhibit enzymatic degradation of glycogen. Tubes were chilled on ice and the worms were grinded with a disposable pestle and aggregated protein and other insoluble material separated by centrifugation. A Glycogen Assay Kit (Sigma, MAK016-1KT) was used to determine glycogen content in the supernatant. As most of the proteins precipitate after boiling, the pellets were dissolved in M9 with 8 M Guanidine HCl, separated from insoluble material and the amount of protein in the supernatant was determined. The total amount of glycogen reported in the Figures was normalized relative to the total protein in the sample. All experiments were repeated at least three times and the average±s.d. presented in the Figures. The detailed results from individual experiments are presented in Supplementary Table 3.

### Determination of reduced thiol content

Total cellular reduced thiols were quantified by reaction with 5,5′-dithiobis-(2-nitrobenzoic) acid) (DTNB)^61^. Worms were allowed to develop and grow on agar plates at 20 °C until L4 and then transferred to NGM plates with or without glucose. For each experimental condition, 100 worms were collected in micro-centrifugal tubes, washed quickly 3 times with M9 buffer and incubated for 10 min at room temperature in M9 buffer with or without 100 mM diamide. All animals survive such short treatment with diamide. Worms were washed three times to remove diamide. After removing most of the liquid, the worms were flash frozen in liquid nitrogen. For analysis, ∼150 μl of M9 buffer with 2 mM DTNB was added to the tubes with worms, frozen animals grinded with a disposable pestle and lysed by two freeze–thaw cycles. Insoluble materials were removed by centrifugation and lysates were clarified using a 10 kDA MWCO spin filter. Absorbance at 412 nm was determined in the flow-through. Thiol concentration in the samples was calculated according to a standard curve generated by reaction of DTNB with GSH and then adjusted to a protein concentration in the lysates.

### Fluorescent reporter assays

Worms expressing GFP under control of *sod-3* (CF1553 and CF1580) were fed on NGM plates seeded with *E. coli* HT115 harbouring either an empty vector or a plasmid-expressing double-stranded *gsy-1* RNAi. One (A1) and 3 (A3) day old adult worms were anaesthetized in a drop of 2% sodium azide and images were captured immediately using a Zeiss AxioZoom v16 microscope equipped for fluorescence illumination. Fluorescence intensity was quantified using the Zeiss ZEN software package.

### Glycogen staining in vivo

To visualize glycogen, live worms were stained over a can with iodine as described in ref. 62. A few worms from control and glucose-supplemented plate were quickly transferred to a fresh plate, which was immediately inverted and placed over a 100 g bottle of iodine for 2 min. Images were produced with a fixed exposure time using a Zeiss Discovery stereomicroscope equipped with colour camera (Optixcam). The mirror on the illuminator stage was adjusted to maximize the worm’s transparency for better visualization of glycogen.

### Lipid staining

Oil-Red-O staining was performed essentially as described in ref. 9. Three-day-old worms were picked, washed with M9 and fixed in 60% isopropanol in PBS. Oil-Red-O stock (Sigma, O1391) was diluted with water to 60% and the precipitate was removed by 0.2 μm filter. Worms were stained for 20 min at room temperature, washed with M9+0.5% triton X100 and placed on NGM plates and images were captured using a Zeiss AxioZoom v16 microscope.

### ATP content

ATP Colorimetric/Fluorometric Assay Kit (Sigma, MAK190-1KT) was used to determine ATP concentration in worm lysates. About 100 worms were picked, washed with M9 buffer and flash frozen in liquid nitrogen. Pellets were grinded with pestles in 100 μl of lysis buffer and diluted with 200 μl of the lysis buffer; cells were lysed by two freeze–thaw cycles and deproteinized using a 10 kDA MWCO spin filter. ATP concentration was measured in the flow-through.

### Cell culture, transfection and glycogen staining

HepG2 cells (ATCC, HB-8065) were grown in DMEM supplemented with 10% (v/v) foetal bovine serum and 10 mM glutamine, pH 7.4. Cells were grown to 70–80% confluence in 100-mm dishes and then transiently transfected using nlucleofection (Lonza) with siRNA-targeting GYS2 following the manufacturer’s protocol. Control cells were transfected with non-targeting siRNA (SiNt). Each siRNA transfection was performed with a cocktail of two siRNAs. And two combinations were used to ensure specificity (Si (2+3), Si (1+3)). For the list of SiRNA see Supplementary Table 5. Transfection efficiency exceeded 85%. Forty-eight hours post-transfection, cells were starved for 2 h in foetal bovine serum-free DMEM before adding media with 12.5 mM glucose. Twenty-four hours after low glucose treatment, the media was replaced by DMEM containing 2 5mM glucose, and recombinant human insulin was added to a final concentration of 25 μM.

Glycogen in hepatocytes was stained by Periodic acid-Schiff (PAS) according to the manufacturer’s protocol (Sigma). PAS-stained cells were imaged using EVOS cell imaging system and images were prepared using the Image J software. Percentage of glycogen was quantified using absorbance at 594 nm to measure PAS intensity and by normalizing it to the intensity of nuclear stain 4,6-diamidino-2-phenylindole at 350 nm.

### Cell culture oxidative stress-resistance assay

HepG2 cells were treated for 30 min with 50 μM H_2_O_2_. The H_2_O_2_-containing media was removed and replaced with fresh media at 37 °C. Cell viability was assayed after 24 h by measuring NucGreen Dead 488 incorporation and fluorescence intensity was normalized to Hoechst staining.

### Sequencing and differential expression analysis

Worms were allowed to develop and grow on agar plates at 20 °C until they reached stage L4 and then transferred to NGM plates with or without glucose and incubated for ∼20 h at 20 °C. Approximately, 300 worms were collected and washed in S-buffer, and total RNA was isolated as described in ref. 63. A TrueSeq RNA Sample Preparation Kit v2 (Illumina) was used to prepare 1 μg of total RNA for RNA-seq. Three independent biological replicates were done for each experimental condition. The reference genome and annotation data for *C. elegans* (Ensembl assembly based on WS220 build) were downloaded from the Illumina website (ftp://igenome:G3nom3s4u@ussd-ftp.illumina.com/Caenorhabditis_elegans/Ensembl/WS220/Caenorhabditis_elegans_Ensembl_WS220.tar.gz). To estimate the expression level of transcripts and test for differential expression between different experimental conditions, the Tophat/Cufflinks/Cuffdiff pipeline was used^64^. Briefly, the RNA-seq reads were trimmed of adapter sequences and then mapped to the *C. elegans* transcriptome with the Tophat software package^65^ using the bowtie2 aligner and default parameters. The transcripts were assembled and their abundances were estimated using the Cufflinks package^66^. A statistical test for differential gene expression was performed using the Cuffdiff tool in the Cufflinks package with a *q* value (*P* value adjusted for multiple testing) threshold of 0.05). Analysis and visualization of the differential expression data was performed with the R software package (version 2.15.1) using the cummeRbund library (version 2.0).

### Data availability

RNA-seq data generated in this study have been deposited in the NCBI GEO with the accession code GSE98576.

## Additional information

**How to cite this article:** Gusarov, I. *et al*. Glycogen controls *Caenorhabditis elegans* lifespan and resistance to oxidative stress. *Nat. Commun.* **8,** 15868 doi: 10.1038/ncomms15868 (2017).

**Publisher’s note:** Springer Nature remains neutral with regard to jurisdictional claims in published maps and institutional affiliations.

## Supplementary Material

Supplementary Information

## Figures and Tables

**Table 1:** Summary of lifespan experiments with wt *C. elegans* strains.

***C.elegans*** **strain**	***E. coli*** **strain/RNAi**	**Media/treatment**	**Mean survival, days±s.d.**	***P*** **value**	**Mean increase/decrease, %±s.d.**
N2	OP50	NGM	14.24±1.23		
N2	OP50	NGM+glucose	10.14±1.03	0.00576	−28.75±4.19
N2	OP50	NGM+15 mM diamide	10.33±0.77		
N2	OP50	NGM+15 mM diamide+glucose	12.72±0.75	0.00909	23.62±12.19
N2	OP50	NGM	13.76±1.12		
N2	OP50	NGM+glucose	9.52±1.00	0.00408	−30.82±3.73
N2	OP50	NGM+5 mM diamide	17.19±0.44	0.00389	25.38±8.12
N2	OP50	NGM+5 mM diamide+glucose	18.32±0.82	0.00233	33.47±5.55
N2	OP50	NGM+paraquat	17.18±1.87	0.03874	23.63±0.29
N2	OP50	NGM+paraquat+glucose	14.04±0.65	0.36199	2.29±5.00
N2	OP50	NGM	12.45±0.50		
N2	OP50	NGM+glucose	9.33±0.23	0.00029	−24.98±4.26
N2	OP50	NGM+acetamonophen	12.65±1.36	0.41259	1.53±9.28
N2	OP50	NGM+acetaminophen+glucose	11.25±0.09	0.00732	−9.55±3.43
N2	e.v.	NGM	15.83±0.76		
N2	e.v.	NGM+glucose	12.53±1.24	0.00844	−20.92±5.29
N2	*gsr-1*	NGM	14.03±0.37	0.01043	−11.26±4.87
N2	*gsr-1*	NGM+glucose	10.68±0.66	0.00044	−32.47±4.97
N2	*gspd-1*	NGM	14.3±0.21	0.03819	−10.59±4.08
N2	*gspd-1*	NGM+glucose	9.83±0.54	0.00123	−38.59±0.38
N2	e.v.	NGM	17.14±1.45		
N2	e.v.	NGM+glucose	12.56±0.77	0.00422	−26.17±9.85
N2	*gsy-1*	NGM	19.66±0.3	0.02129	15.23±10.22
N2	*gsy-1*	NGM+glucose	15.41±0.62	0.06468	−9.78±7.17
N2	*pyg-1*	NGM	18.03±1.49	0.25163	5.7±13.41
N2	*pyg-1*	NGM+glucose	11.54±0.27	0.00138	−32.32±6.32
N2	e.v.	NGM+5 mM diamide+glucose	18.13±1.45		
N2	*gsy-1*	NGM+5 mM diamide+glucose	18.41±0.71	0.38957	1.77±4.61
N2	*pyg-1*	NGM+5 mM diamide+glucose	15.01±0.34	0.01113	−16.96±5.04

The percentage of increase (+) or decrease (−) of the lifespan is shown relative to the control of the same experiment. *P* values were calculated with respect to control animals in the same experiment using Student’s *t*-test (one-tailed distribution and two-sample equal variance). See also Supplementary Table 2 for detail on individual experiments.

**Table 2:** Summary of lifespan experiments with mutant *C. elegans* strains.

***C.elegans*** **strain**	***E. coli*** **strain/RNAi**	**Media/treatment**	**Mean survival, days±s.d.**	***P*** **value**	**Mean increase/decrease, %±s.d.**
*daf-2*	e.v.	NGM	34.83±0.35		
*daf-2*	e.v.	NGM+glucose	29.29±0.46	0.00004	−15.91±1.16
*daf-2*	*gsy-1*	NGM	32.86±0.73	0.00678	−5.66±2.22
*daf-2*	*gsy-1*	NGM+glucose	32.95±1.31	0.03744	−5.41±3.09
*daf-2*	*pyg-1*	NGM	37.34±3.86	0.16279	7.15±10.43
*daf-2*	*pyg-1*	NGM+glucose	21.66±1.03	0.00002	−37.83±2.37
*daf-16*	e.v.	NGM	12.35±1.2		
*daf-16*	e.v.	NGM+glucose	9.55±0.49	0.01001	−22.21±8.2
*daf-16*	*gsy-1*	NGM	13.95±0.88	0.06641	−13.35±8.3
*daf-16*	*gsy-1*	NGM+glucose	10.79±0.23	0.04679	−12.17±8.16
*daf-2; daf-16*	e.v.	NGM	13.15±1.24		
*daf-2; daf-16*	e.v.	NGM+glucose	11.53±0.72	0.03867	−12.24±0.26
*daf-2; daf-16*	*gsy-1*	NGM	14.56±1.16	0.11200	11.02±8.25
*daf-2; daf-16*	*gsy-1*	NGM+glucose	12.28±0.19	0.10455	−6.18±8.50
*aak-2*	e.v.	NGM	12.76±0.16		
*aak-2*	e.v.	NGM+glucose	10.23±0.55	0.00076	−19.85±3.35
*aak-2*	*gsy-1*	NGM	12.54±0.77	0.32458	−1.79±4.87
*aak-2*	*gsy-1*	NGM+glucose	9.89±0.32	0.00008	−22.53±2.5

The percentage of increase (+) or decrease (−) of the lifespan is shown relative to the control of the same experiment. *P* values were calculated with respect to control animals in the same experiment using Student’s *t*-test (one-tailed distribution and two-sample equal variance). See also Supplementary Table 2 for detail on individual experiments.
