# Supplementary material for: Glycogen controls Caenorhabditis elegans lifespan and resistance to oxidative stress
Source: Nat Commun. 2017 Jun 19;8:15868. doi: 10.1038/ncomms15868 (PMC5481799; doi:10.1038/ncomms15868)
Supplement: Supplementary Information [file ncomms15868-s1.pdf]

Type of file: pdf
Size of file: 0 KB
Title of file for HTML: Supplementary Information
Description: Supplementary Figures, Supplementary Tables.

SUPPLEMENTARY FIGURES

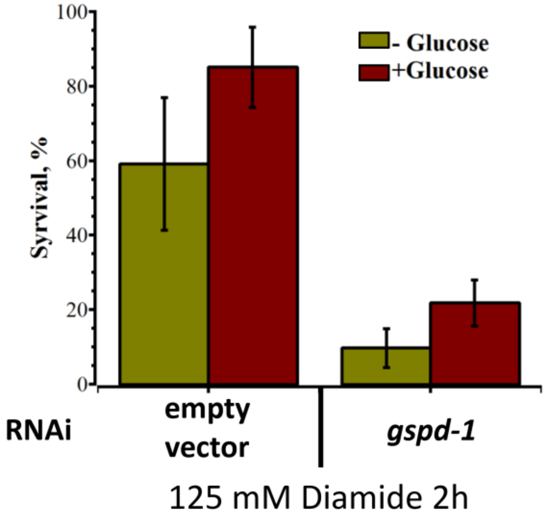

Supplementary Figure 1. **Depletion of glucose-6-phosphat dehydrogenase (*gspd-1*) by RNAi sensitizes *C. elegans* to 125 mM diamide**. The graph shows the average survival ±SD of at least three independent experiments (e.v.: n=130, *p*=0.036; *gspd-1*: n=135 *p*=0.02). Experimental conditions were the same as in Fig. 2b except 125 mM diamide was used. A small amount of NADPH can be generated by the malic enzyme[67], which can explain a modest increase in diamide resistance in *gspd-1*-deficient worms fed a high glucose diet.

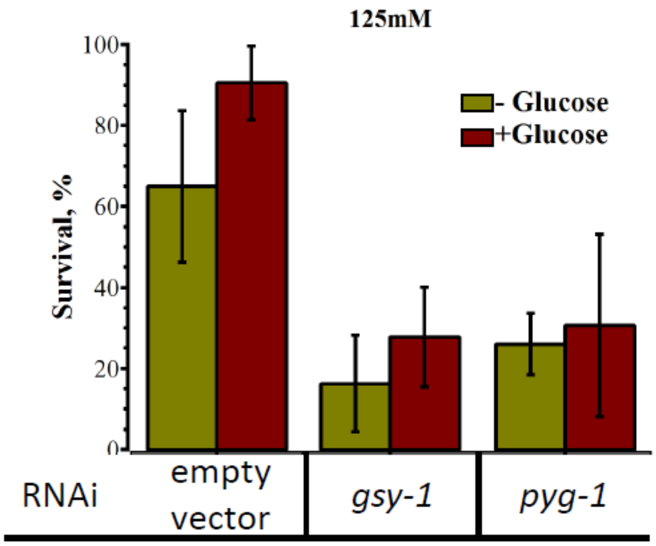

Supplementary Figure 2. Glycogen storage is required for protection against oxidants. N2 worms fed on NGM plates seeded with *E. coli* HT115 harboring either empty plasmid vector or plasmid vectors expressing *gsy-1* or *pyg-1* RNAi. L4 stage animals were transferred to NGM plates with sorbitol (green bars) or with glucose (red bars) for 20 hours and then treated with 125 mM diamide. The graph shows the average survival ±SD of at least three independent experiments (e.v.: n=135, p=0.024; *gsy-1*: n=190 p=0.05; *pyg-1*: n=95 p= 0.376).

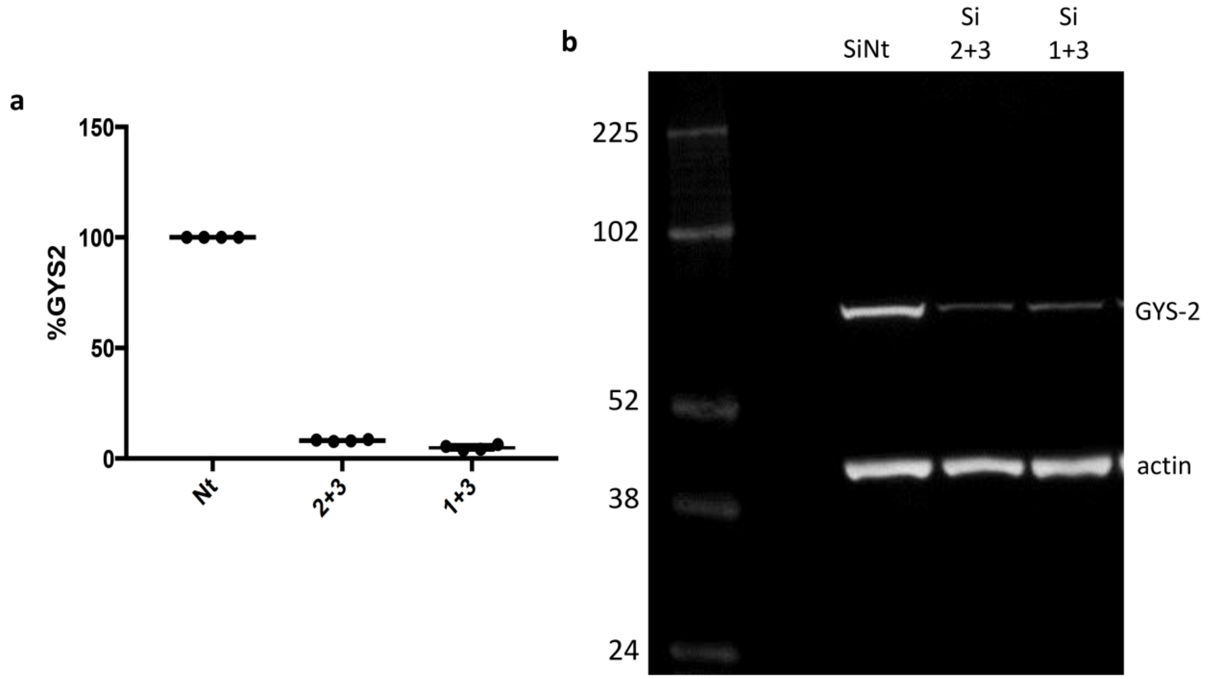

Supplementary Figure 3. Glycogen synthase 2 knockdown blocks glycogen synthesis in hepatocytes. (**a**) RT-qPCR quantitation of GYS2 knockdown. Percent of GYS2 RNA remaining in HepG2 cells was calculated with respect to Nt. Nt - Non target siRNA. (2+3) - Combination of SiRNA 2 and 3. (1+3) - Combination of siRNA 1 and 3. Values of four independent experiments are plotted. (**b**) Western blot analysis of GYS2 knockdown in HepG2 cells.

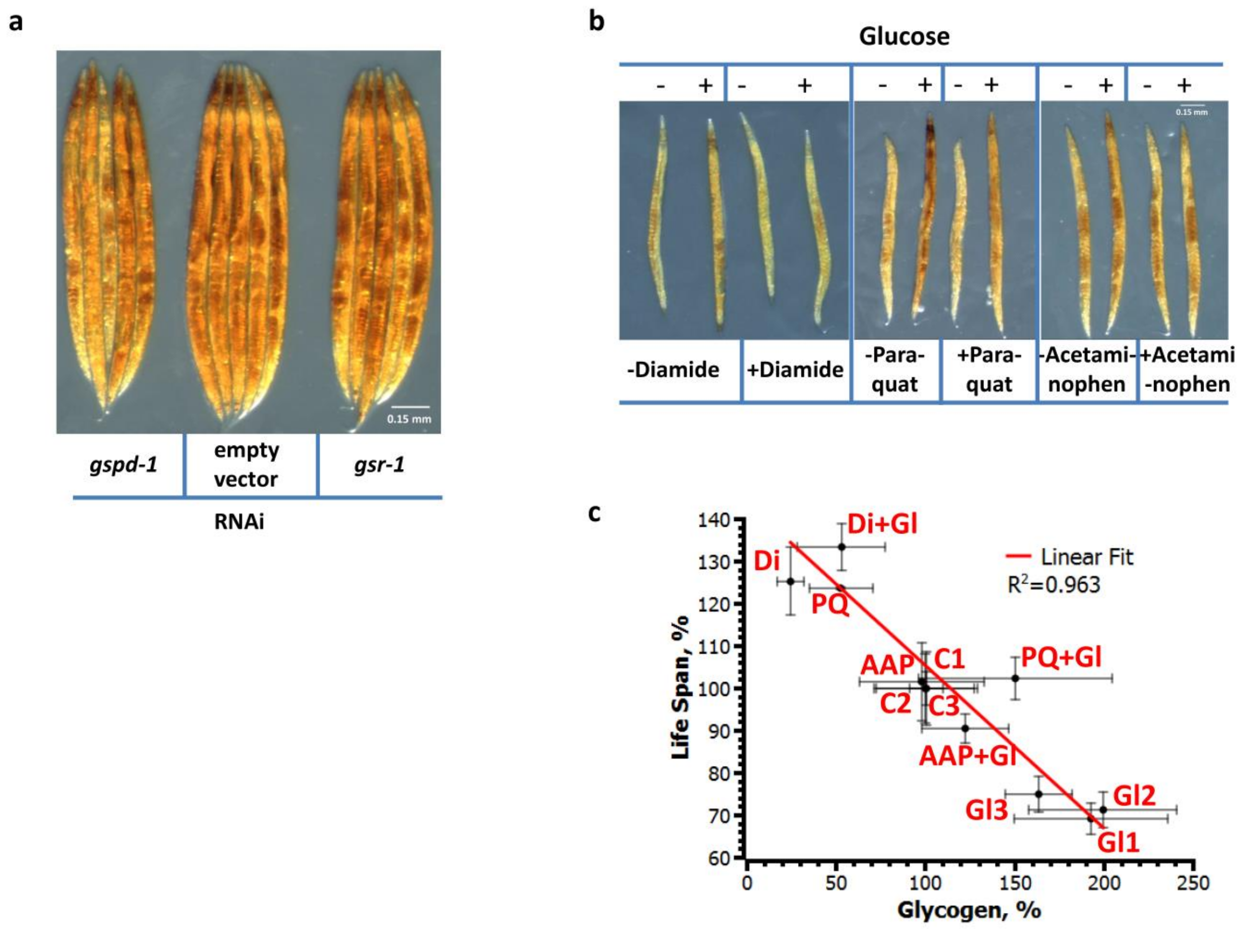

Supplementary Figure 4. Glycogen staining in live *C. elegans* and correlation between life span and glycogen level. Images were captured with a color camera while the mirror in the illumination stage was adjusted to maximize the worm's transparency. (**a**) Representative images of three-day old *gsr-1* and *gspd-1* RNAi treated worms stained for glycogen with iodine are shown (see Material and Methods for details). (**b**) Representative images of three-day old wt worms stained for glycogen with iodine are shown. Experimental conditions were the same as in Fig. 4. (c) Lifespan has an inverted linear relationship with glycogen accumulation. The Y-axis represents changes of the lifespan (%, from Supplementary Table 2) in the experimental group (NGM media + reagents) versus the control group (NGM media, 100% lifespan). The X-axis represents changes in the glycogen content (%, from Supplementary Table 3) in the experimental group (NGM media + reagents) vs. the control group (NGM media, 100% glycogen). Reagents: glucose (Gl), diamide (Di), paraquat (PQ), or acetaminophen (AAP). C- control, no reagent added. A linear fit of the data was accomplished using SciDavis software.

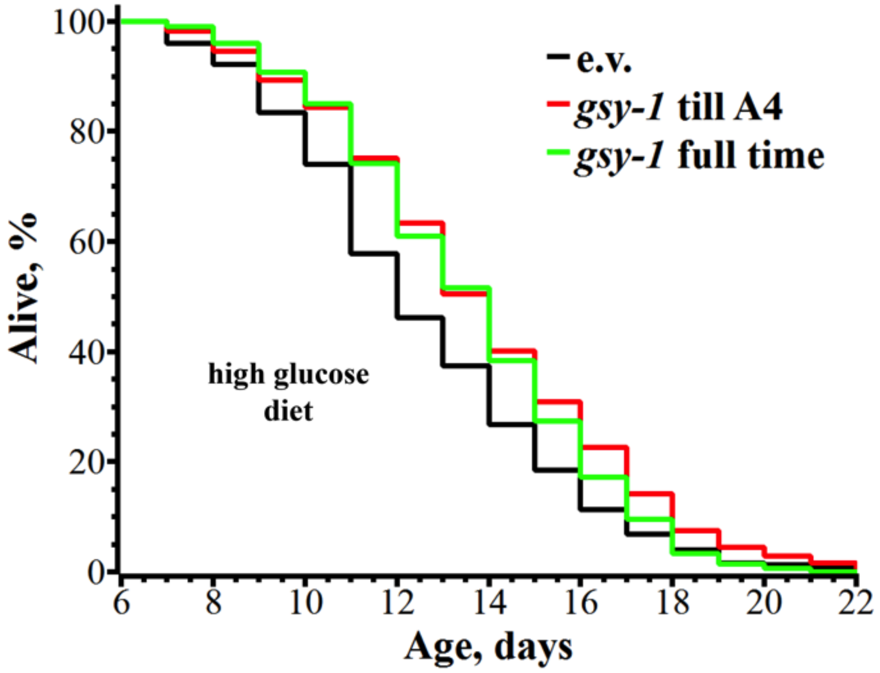

Supplementary Figure 5. On a high glucose diet, glycogen depletion until day four of adulthood is as efficient for the lifespan extension as a life-long *gsy-1* RNAi treatment. N2 worms fed on NGM plates seeded with *E. coli* HT115 harboring either empty plasmid vector or plasmid vector expressing *gsy-1* RNAi. L4 animals were transferred on NGM plates with or without glucose. At day 4 of adulthood half of *gsy-1* RNAi fed worms were transferred on NGM plates seeded with *E. coli* HT115 harboring empty plasmid vector. The graph shows the average of three independent experiments on a high glucose diet (see also supplementary Table 2).

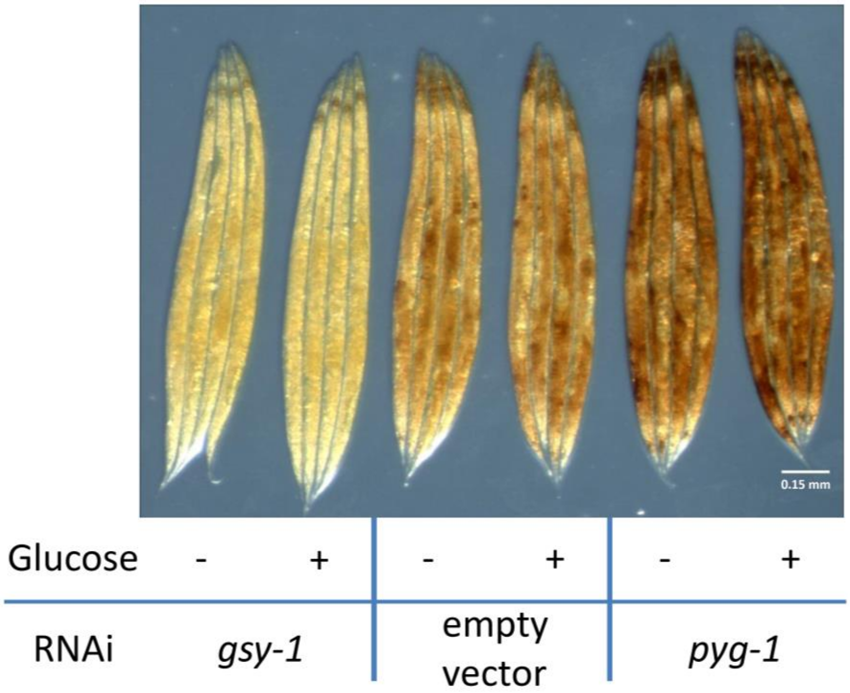

Supplementary Figure 6. The effect of glycogen synthase (*gsy-1*) or glycogen phosphorylase (*pyg-1*) depletion on glycogen content of *daf-2* worms. Representative images of seven-day old worms stained with iodine are shown (see Material and Methods for details). Experimental conditions were the same as in Fig. 7. Images were captured with a color camera while the mirror in the illumination stage was adjusted to maximize the worm's transparency.

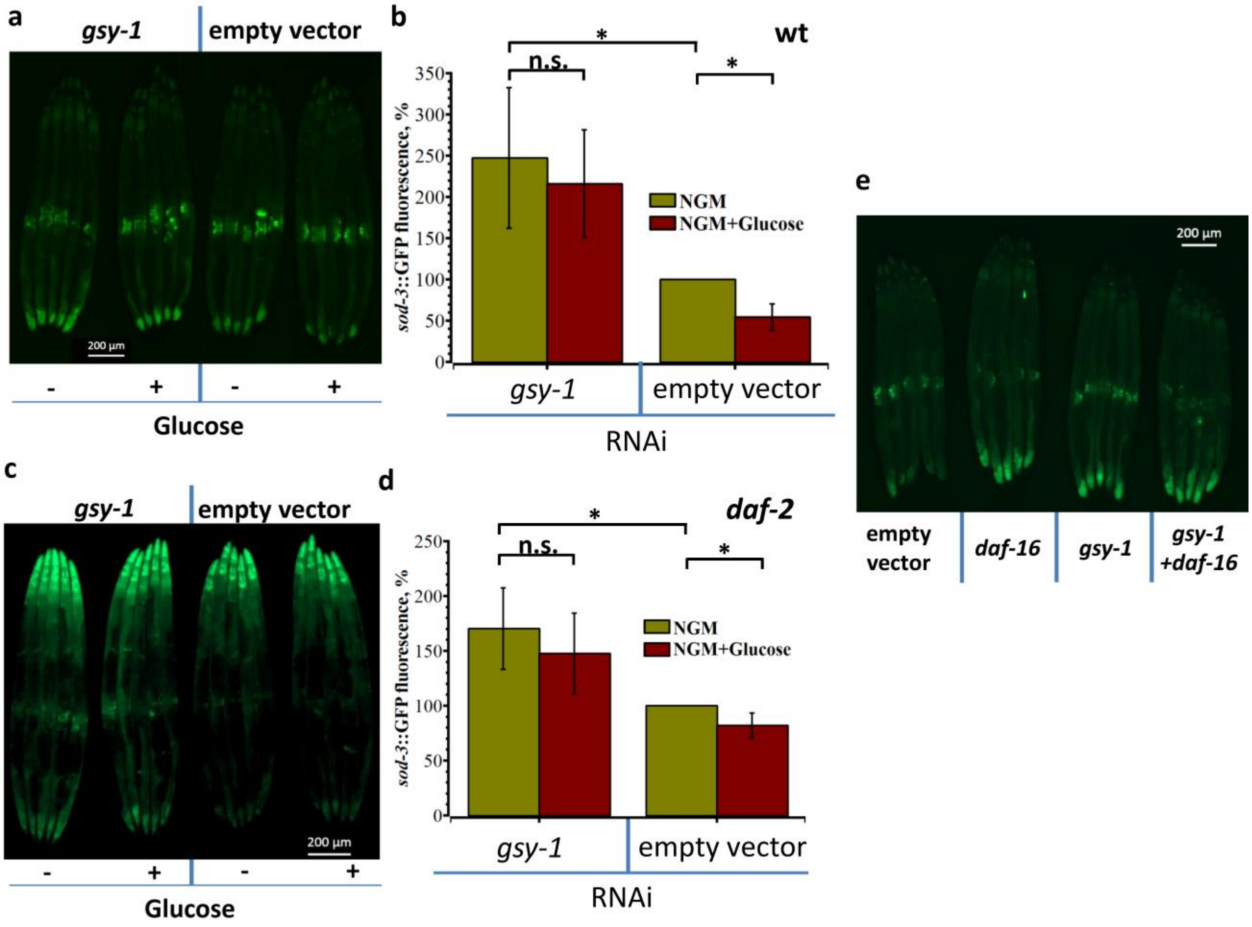

Supplementary Figure 7. Glycogen inhibits SOD-3 expression independently of DAF-16 and IIS. Glycogen depletion upregulates SOD-3 transcription in wt (**a** and **b**) and *daf-2* (**c** and **d**) *C. elegans*. Representative fluorescent image (a and c) and quantification (**b** and **d**) of three days old adult worms expressing GFP under control of *sod-3* promoter (CF1553 and CF1580 strains). Animals were fed on NGM or NGM+Glucose plates seeded with *E.coli* HT115 harboring either an empty vector or a plasmid expressing double-stranded *gsy-1* RNAi. Graphs in (**b** and **d**) show the relative level of fluorescence in the tails of experimental group compared to control group. Data from three independent experiments are presented as the mean ± SD (*P<0.05), and at least 50 worms per condition pooled together. n.s. – non significant. See also Supplementary Table 4. (**e**) *daf-16* RNAi upregulate tail *sod-3*::GFP expression. Representative imajes of wt worms fed *E.coli* HT115 harboring either an empty vector, *daf-16*, *gsy-1* or *daf-16 gsy-1* double RNAi are shown.

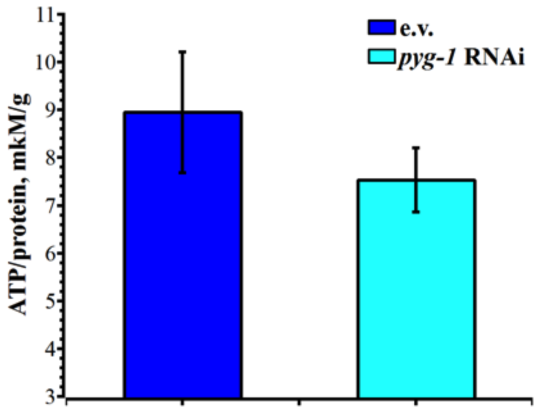

Supplementary Figure 8. ATP level in wt and glycogen phosphorylase (*pyg-1*)-deficient worms. Depletion of glycogen phosphorylase traps cellular glucose and decreases its availability for ATP production. Wt worms were fed on NGM plates seeded with *E.coli* HT115 harboring either an empty vector (blue) or a plasmid expressing either double-stranded *pyg-1* RNAi (cyan). One day old worms were collected, washed and ATP concentration determined in worm lysates. The results from three independent experiments ± SD are presented (n=400, p=0.048).

Supplementary Table 1. *C. elegans* genes differentially regulated by glucose diet

Gene short name	NGM	NGM+ Glu-cose	Fold cha-nge	q-value	Cellular process	Putative function*
**cnc-1**	0.00	1.92	Inf	0.0047	immune response, high salt	antimicrobial peptide canaecin
**ilys-3**	0.00	5.24	Inf	0.0047	immune response	lysozyme
**spp-21**	0.00	1.41	Inf	0.0144	osmolarity	SaPosin-like Protein family
**Y102A5C.34**	0.00	5.00	Inf	0.0047	unknown	GRounDhog (hedgehog-like family)
**F48C1.9, spe-11**	2.71	297.12	6.78	0.0047	multicellular organism development	protein required for early embryonic development and sperm pseudopods
**cpt-3**	0.07	2.68	5.31	0.0254	lipid metabolism	carnitine palmitoyltransferase
**spp-12**	1.47	50.93	5.11	0.0047	determination of adult lifespan	a saposin-like caenopore; required for the defense response
**far-3**	46.28	984.01	4.41	0.0047	lipids metabolism	Fatty Acid/Retinol binding protein
**nlp-25**	4.52	59.69	3.72	0.0047	neuropeptide signaling pathway	Neuropeptide-Like Protein
**srsx-36, srsx-38**	0.13	1.71	3.69	0.0087	signaling	Serpentine Receptor, class SX
**ora-1**	0.33	4.00	3.61	0.0087	unknown	Onchocerca Related Antigen family
**F09F3.8**	0.25	2.86	3.50	0.0047	unknown	
**F12A10.1**	30.96	292.90	3.24	0.0047	unknown	
**cyp-13B1**	0.29	2.36	3.03	0.0047	oxidation reduction	cytochrome P450
**C32D5.12**	2.04	15.66	2.94	0.0047	lipids metabolism	3-beta-hydroxy-delta5-steroid dehydrogenase, body morphogenesis, development, locomotion, and reproduction
**fat-5**	14.27	106.24	2.90	0.0047	lipids metabolism	delta-9 fatty acid desaturase
**ugt-14**	0.18	1.29	2.82	0.0254	glucose metabolism	UDP glycosyltransferase
**ptr-22**	0.17	1.13	2.69	0.0144	structural protein/collagen/cuticle	the sterol sensing domain (SSD) protein
**amt-1**	3.68	22.02	2.58	0.0047	ammonium metabolism	ammonium ions transporter
**T02B11.4**	1.88	10.79	2.52	0.0047	unknown	
**F57H12.6**	0.56	3.07	2.47	0.0498	unknown	
**M162.5**	1.01	5.34	2.41	0.0047	transport	Solute carriers family
**ZK970.7**	3.23	15.94	2.30	0.0047	unknown	Onchocerca volvulus Ov17 hypodermal antigen; expression regulated by ionizing radiation and bacterial infection
**fmo-2**	0.34	1.69	2.29	0.0116	immune response	a flavin-containing monoxygenase
**far-4**	1.20	5.76	2.26	0.0047	lipids metabolism	a fatty acid and retinol-binding protein, binds lipids in vitro
**dhs-26**	1.25	5.74	2.20	0.0047	lipids metabolism	a short-chain dehydrogenase
**dct-3**	0.64	2.88	2.18	0.0254	unknown	amyotrophic lateral sclerosis 2 chromosome region candidate
**M03A1.8**	0.54	2.33	2.12	0.0254	oxidation-reduction process	ferric chelate reductase 1
**nlp-34**	7.60	32.61	2.10	0.0087	signaling	Neuropeptide-Like Protein
**T19B10.2**	32.55	131.90	2.02	0.0047	structural protein/collagen/cuticle	determine adult lifespan, locomotion, the molting cycle, development and reproduction
**F47C12.1**	0.19	0.76	1.98	0.0087	unknown	
**tsp-1**	4.06	16.00	1.98	0.0047	immune response	Tetraspanin family
**Y41C4A.11**	11.08	42.53	1.94	0.0047	transport	beta' subunit of the coatomer complex
**tsp-2**	4.73	17.78	1.91	0.0047	signaling	CD63
**hmit-1.1**	1.92	7.17	1.90	0.0047	osmolarity	proton (H+)-dependent myo-inositol transporters; potentially regulate cell signaling and intracellular

						osmolarity.
**T26H5.9**	50.24	187.47	1.90	0.0047	unknown	
**F10D2.10,fat-7**	73.89	274.22	1.89	0.0047	lipids metabolism	delta-9 fatty acid desaturase that is required for the synthesis of monounsaturated fatty acids
**C02E7.10**	3.85	14.17	1.88	0.0047	unknown	
**Y77E11A.12**	1.62	5.79	1.83	0.0047	membrane	GRAMD4
**T22B7.3**	2.80	9.89	1.82	0.0047	unknown	
**nhr-11**	2.33	8.08	1.79	0.0047	transcription factor	superfamily of nuclear receptors
**oac-14**	4.46	15.17	1.77	0.0047	lipids metabolism	O-Acyltransferase, Cd++ sensitive, slow growth
**cutl-2**	1.31	4.34	1.73	0.0047	structural protein/collagen/cuticle	CUTiclin-Like
**ugt-57**	0.61	2.00	1.72	0.0144	glucose metabolism	transfer hexosyl groups
**ces-2**	2.73	8.87	1.70	0.0047	transcription factor	leucine-zipper (bZIP) transcription factor, most similar in sequence and binding specificity to the PAR (proline- and acid-rich) subfamily of bZIP proteins and is required to activate programmed cell death
**bcmo-1**	0.73	2.36	1.69	0.0116	lipids metabolism	retinal pigment epithelium-specific protein and beta-carotene oxygenase
**dhs-5**	4.82	15.53	1.69	0.0047	lipids metabolism	steroid dehydrogenase
**lys-3**	5.93	18.70	1.66	0.0047	unknown	lysozyme
**T12D8.5,arrd-17**	31.15	97.48	1.65	0.0047	signaling	Alpha arrestins family, binds phosphatase 2B
**gpdh-1**	5.80	17.72	1.61	0.0350	glucose metabolism	glycerol 3-phosphate dehydrogenases
**C05D11.5,nas-4**	8.67	26.14	1.59	0.0047	glucose metabolism	hydroxypyruvate isomerase
**cyp-13A10**	0.94	2.73	1.54	0.0116	oxidation-reduction process	cytochrome P450s
**F59A7.2**	5.47	15.73	1.52	0.0174	unknown	
**cyp-25A3**	6.52	18.65	1.52	0.0047	lipids metabolism	thromboxane A synthase 1
**dod-3**	15.93	45.49	1.51	0.0047	determination of adult lifespan	determination of adult lifespan
**mlt-11**	1.17	3.35	1.51	0.0047	development	Tissue Factor Pathway Inhibitors (TFPIs), serine protease inhibitors that function in blood coagulation and required for proper molting
**D2063.1**	1.64	4.64	1.50	0.0116	oxidation-reduction process	putative oxidoreductase
**hch-1**	2.85	8.02	1.49	0.0047	protease	zinc-dependent metalloprotease
**C53A3.2**	17.21	47.11	1.45	0.0047	glucose metabolism	phosphoglycolate phosphatase and pyridoxal (pyridoxine, vitamin B6) phosphatase
**W02C12.1**	0.48	1.32	1.45	0.0144	unknown	
**tag-38**	3.38	9.21	1.45	0.0047	lipids metabolism	sphingosine-1-phosphate lyase
**F56C11.6**	3.27	8.86	1.44	0.0047	development	embryo development
**Y41G9A.10**	7.67	20.76	1.44	0.0047	unknown	
**cyp-32A1**	3.58	9.56	1.42	0.0047	oxidation-reduction process	heme binding activity and oxidoreductase activity
**C50F7.5**	3.98	10.60	1.41	0.0047	unknown	
**F44A2.3**	3.80	10.03	1.40	0.0047	lipids metabolism	bactericidal/permeability-increasing protein and lipopolysaccharide binding protein, is involved in embryo

						development and is predicted to have lipid binding activity
**R09E10.5**	0.74	1.94	1.39	0.0144	membrane	
**cyp-29A2**	7.25	18.98	1.39	0.0047	lipids metabolism	cytochrome P450 family, involved in lipid storage
**egl-46**	2.22	5.74	1.37	0.0279	transcription factor	transcription factor with similarity to the TFIIA and is required for coordinated locomotion, morphology and process formation of the touch cells, male mating efficiency, HSN cell migration, differentiation, and axonal outgrowth, serotonin production and also terminal divisions of the Q neuroblasts
**F33H12.7**	19.71	50.65	1.36	0.0047	unknown	dauer lifespan extended
**F53A9.2**	7.26	18.17	1.32	0.0472	metal stress response	is required for the stress response to copper ion, FOS-1and kgb-1-dependent
**nhr-43**	1.78	4.42	1.31	0.0087	transcription factor	nuclear hormone receptor
**T02E9.5**	15.60	38.48	1.30	0.0047	unknown	
**dao-2**	26.21	63.82	1.28	0.0047	unknown	secreted protein with a DB module, may be involved in dauer formation.
**C09F9.2**	1.50	3.64	1.28	0.0087	membrane	
**21ur-11897,noah-2**	7.24	17.49	1.27	0.0047	structural protein/collagen/cuticle	PAN and ZP domain-containing protein is essential for molting, embryonic and larval development, reproduction, coordinated locomotion, and the overall health of the animal.
**gln-3**	56.89	137.29	1.27	0.0047	ammonium metabolism	glutamate-ammonia ligase
**acl-12**	10.66	25.44	1.26	0.0047	lipids metabolism	lysophosphatidylglycerol acyltransferase, involved in lipid storage
**R10D12.9**	26.10	61.87	1.25	0.0047	glucose metabolism	SWEET sugar transporter family
**cpt-5**	8.18	19.21	1.23	0.0047	lipids metabolism	carnitine palmitoyltransferase
**lact-5**	3.79	8.87	1.23	0.0047	unknown	beta-lactamase domain-containing proteins.
**sqt-3**	11.05	25.59	1.21	0.0047	structural protein/collagen/cuticle	cuticle collagen
**Y87G2A.2**	15.88	36.67	1.21	0.0199	lipids metabolism	acyl-CoA thioesterase 8
**T22B7.7**	15.27	35.11	1.20	0.0047	lipids metabolism	acyl-CoA thioesterase 9
**Y73C8B.1**	3.52	8.08	1.20	0.0087	hydrolase	
**ugt-32**	1.93	4.42	1.20	0.0448	glucose metabolism	UDP glycosyltransferase family
**C08E8.4**	10.67	24.31	1.19	0.0047	immune response	innate immune response
**alh-5**	3.49	7.95	1.19	0.0047	oxidation-reduction process	Aldehyde dehydrogenases family
**ugt-16**	4.65	10.54	1.18	0.0047	glucose metabolism	UDP glycosyltransferase family
**ptr-14**	4.72	10.68	1.18	0.0047	structural protein/collagen/cuticle	sterol sensing domain proteins, required for normal growth to full size and locomotion.
**F53A9.8**	21.80	49.24	1.18	0.0047	immune response	involved in defense response to Gram-positive bacterium
**fmo-3**	7.11	15.98	1.17	0.0047	oxidation-reduction process	flavin-containing monoxygenase homologous to human FMO1, FMO2, and FMO3
**dpy-14**	27.26	60.63	1.15	0.0047	structural protein/collagen/cuticle	type III (alpha 1) collagen that is required for embryonic, larval, and vulval development, proper amphid morphology, and regulation of body shape and size

**R07E3.4**	21.64	47.95	1.15	0.0047	transport	solute carrier family 25 member 31
**wrt-10**	7.20	15.88	1.14	0.0116	signaling	hedgehog-like protein, involved in intercellular signalling and is required for normal growth to full size and locomotion
**C15C8.3**	143.57	313.20	1.13	0.0397	protease	napsin A aspartic peptidase, renin and cathepsin D
**ptr-8**	5.99	13.03	1.12	0.0047	structural protein/collagen/cuticle	sterol sensing domain protein, required for normal growth to full size and locomotion
**Y37H2A.13, Y37H2A.14**	24.02	51.61	1.10	0.0047	membrane	
**F58F9.7**	16.33	34.94	1.10	0.0047	lipids metabolism	acyl-CoA oxidase 3 which responcible for desaturation of 2-methyl branched fatty acids in peroxisomes
**noah-1**	7.95	16.82	1.08	0.0047	structural protein/collagen/cuticle	PAN and ZP domain-containing protein that required for embryonic and larval development, vulval development, coordinated locomotion, and normal body morphology.
**F41C3.2**	3.48	7.34	1.08	0.0116	transport	Solute carriers family
**B0272.4**	14.46	30.25	1.06	0.0047	transcription factor	chromodomain protein
**F44E7.2**	32.13	67.14	1.06	0.0047	glucose metabolism	phosphoglycolate phosphatase and pyridoxal (pyridoxine, vitamin B6) phosphatase
**cnc-4**	18.47	38.16	1.05	0.0199	immune response	caenacin strongly induced after infection by the fungus
**thn-2**	23.02	47.52	1.05	0.0047	immune response	involved in defense response
**nlp-29**	68.21	140.60	1.04	0.0047	immune response	antimicrobial, neuropeptide-like protein
**Y53G8B.2**	17.15	35.10	1.03	0.0047	lipids metabolism	monoacylglycerol O-acyltransferase 1, diacylglycerol O-acyltransferase 2 and monoacylglycerol O-acyltransferase 2
**dpy-17**	29.54	60.14	1.03	0.0047	structural protein/collagen/cuticle	cuticle collage
**hrg-4**	14.10	28.45	1.01	0.0304	heme binding	transmembrane protein that is mediate heme homeostasis
**21ur-13376,Y105C5A.8**	31.29	62.62	1.00	0.0047	unknown	
**fmi-1**	0.59	1.18	1.00	0.0350	signaling	cadherin-like protein, which plays a crucial role in pioneer axon navigation
**hmit-1.2**	3.25	6.47	0.99	0.0199	lipids metabolism	proton (H+)-dependent myo-inositol transporters which potentially regulate cell signaling and osmolarity
**ckb-2**	34.81	69.17	0.99	0.0047	lipids metabolism	choline kinase
**ugt-25**	7.92	15.45	0.96	0.0087	glucose metabolism	UDP glycosyltransferase family 3
**aqp-2**	32.30	62.84	0.96	0.0047	transport	aquaglyceroporin
**F08B12.4**	143.83	279.13	0.96	0.0047	unknown	
**aqp-8**	34.17	65.92	0.95	0.0047	transport	aquaglyceroporin
**K10D11.5**	13.87	26.72	0.95	0.0047	unknown	epoxide hydrolase 1
**dpy-10**	5.25	10.10	0.94	0.0199	structural protein/collagen/cuticle	A cuticle collagen protein that affects body morphology and movement
**mnp-1**	4.73	9.09	0.94	0.0087	protease	M1 family of metalloproteinases
**alh-10**	19.05	36.52	0.94	0.0047	oxidation-reduction process	aldehyde dehydrogenase 8 family

**tts-1**	56.27	107.66	0.94	0.0047	unknown	Transcribed Telomerase-like Sequence
**Y75B8A.4**	8.21	15.56	0.92	0.0047	protease	lon peptidase 2, peroxisomal
**F10E9.1,F10E9.12**	14.31	26.84	0.91	0.0350	unknown	
**Y73B6BL.31**	35.41	65.85	0.89	0.0047	transport	solute carrier family 35
**dhs-18**	27.11	50.17	0.89	0.0047	lipids metabolism	a predicted short-chain dehydrogenase.
**F59A1.10**	25.95	47.53	0.87	0.0047	lipids metabolism	monoacylglycerol O-acyltransferases and diacylglycerol O-acyltransferase 2
**F54F3.4**	16.58	29.67	0.84	0.0116	oxidation-reduction process	dehydrogenase/reductase family
**Y48A6B.9**	7.99	14.16	0.83	0.0350	lipids metabolism	mitochondrial trans-2-enoyl-CoA reductase
**clec-5**	25.28	44.66	0.82	0.0047	carbohydrate binding	C-type lectin domain family 3
**ttr-51**	657.57	1,153.75	0.81	0.0448	unknown	TransThyretin-Related family domain
**lam-3**	3.01	5.26	0.81	0.0047	development	laminin A, a basement membrane component required for normal tissue development, including pharyngeal development
**F23F12.12**	21.42	37.42	0.80	0.0279	unknown	
**ech-8**	12.07	21.05	0.80	0.0304	lipids metabolism	enoyl-CoA, hydratase/3-hydroxyacyl CoA dehydrogenase, involved in lipid storage
**ZK550.6**	36.64	63.89	0.80	0.0047	lipids metabolism	PHYTANOYL-COA HYDROXYLASE
**W07B8.1**	22.35	38.74	0.79	0.0116	protease	cathepsin B, peptidase
**sto-1**	15.93	27.45	0.79	0.0279	structural protein/collagen/cuticle	nephrosis 2, idiopathic, steroid-resistant (podocin)
**fbxc-51**	12.52	21.53	0.78	0.0230	unknown	
**clec-266**	62.24	106.44	0.77	0.0144	signaling	C-type lectin domain family
**acs-17**	10.95	18.65	0.77	0.0144	lipids metabolism	acyl-CoA synthetase long-chain family, determine adult lifespan and embryo development
**T01D1.4**	28.32	48.23	0.77	0.0254	methionine metabolism	acireductone dioxygenase 1, involved in embryo development
**M02H5.8**	94.80	160.26	0.76	0.0174	unknown	
**T28C12.4**	15.48	26.11	0.75	0.0047	structural protein/collagen/cuticle	neuroligin 4
**dhs-19**	32.37	54.38	0.75	0.0174	lipids metabolism	short-chain dehydrogenase
**F45D3.4**	38.42	64.54	0.75	0.0199	unknown	
**F53B3.5**	15.94	26.75	0.75	0.0279	membrane	claudin that may regulate ion channels
**21ur-13425,C17H12.8**	114.82	192.15	0.74	0.0174	immune response	
**cht-1**	38.47	64.32	0.74	0.0047	structural protein/collagen/cuticle	chitinase-1
**dhs-28**	102.12	169.95	0.73	0.0047	lipids metabolism	17-BETA-HYDROXYSTEROID DEHYDROGENASE 4
**asns-2**	21.11	34.99	0.73	0.0047	ammonium metabolism	asparagine synthetase (glutamine-hydrolyzing)
**Y57A10A.23**	46.25	75.93	0.72	0.0199	cell differentiation	anterior gradient 3, protein disulphide isomerase family member
**F08A8.2,acox-1**	28.87	47.38	0.71	0.0116	lipids metabolism	ACYL-CoA OXIDASE 1, PALMITOYL
**W01C8.5**	18.65	30.59	0.71	0.0144	unknown	thrombomodulin
**sodh-1**	25.31	40.98	0.70	0.0397	glucose metabolism	determine adult lifespan, have oxidoreductase activity

**ugt-44**	30.83	49.79	0.69	0.0116	immune response	UDP glycosyltransferase family 3
**W01B11.6**	99.26	160.23	0.69	0.0397	thioredoxin like	disulfide oxidoreductase activity
**C27B7.9,rap-1**	195.35	315.11	0.69	0.0144	unknown	
**R193.2**	11.50	18.49	0.69	0.0230	membrane	
**daf-22**	53.07	85.08	0.68	0.0047	lipids metabolism	sterol carrier protein SCP2, which catalyzes the final step in peroxisomal fatty acid beta-oxidation and is required for dauer pheromone biosynthesis
**his-24**	122.70	195.91	0.68	0.0047	histone	H1 linker histone
**W01F3.2**	55.52	88.57	0.67	0.0116	unknown	
**21ur-14779,C49C8.5**	33.77	53.81	0.67	0.0350	immune response	Fibrinogen C domain containing family including ANGPT2
**T28H10.3**	103.82	164.15	0.66	0.0328	development	legumain, involved in embryo development and reproduction; peptidase activity
**C31C9.2**	61.68	97.03	0.65	0.0144	glucose metabolism	PHOSPHOGLYCERATE DEHYDROGENASE
**cpr-5**	381.35	597.20	0.65	0.0116	protease	cysteine protease.
**cpr-4**	414.10	639.15	0.63	0.0328	protease	cathepsin B-like cysteine protease
**Y71A12C.2, smd-1**	67.41	103.75	0.62	0.0254	Cys/SAM metabolism	S-adenosylmethionine decarboxylase
**cav-1**	159.51	237.48	0.57	0.0472	lipids metabolism	caveolin
**Y69H2.3**	72.88	47.83	-0.58	0.0376	immune response	embryo development, hermaphrodite genitalia development, locomotion and reproduction.
**C23H5.8**	251.07	165.11	-0.60	0.0116	immune response	
**acdh-9**	104.62	66.76	-0.65	0.0087	lipids metabolism	acyl-CoA dehydrogenase family
**spp-3**	629.63	401.62	-0.65	0.0047	immune response	caenopore, similar to saposin-like proteins
**F31F7.1**	80.50	50.73	-0.67	0.0174	immune response	embryo development
**F47B10.2**	29.55	18.58	-0.67	0.0304	ammonium metabolism	histidine ammonia lyase
**lipl-5**	122.02	76.42	-0.67	0.0199	lipids metabolism	lipase
**pcp-2**	8.88	5.39	-0.72	0.0448	protease	prolyl carboxypeptidase
**C45B2.1**	252.10	151.36	-0.74	0.0047	unknown	
**clec-97**	75.64	44.96	-0.75	0.0304	lipids metabolism	lipid storage
**dhs-25**	229.67	135.41	-0.76	0.0116	lipids metabolism	short-chain dehydrogenase
**clec-50**	224.85	131.11	-0.78	0.0047	signaling	Collectins family
**col-95**	278.27	161.95	-0.78	0.0047	structural protein/collagen/cuticle	collagen
**spp-8**	29.08	16.88	-0.79	0.0116	lipids metabolism	prosaposin
**C12C8.2**	39.61	22.79	-0.80	0.0047	Cys/SAM metabolism	cystathionine gamma-lyase
**R03D7.2**	13.27	7.61	-0.80	0.0199	DNA damage response	response to DNA damage
**spp-4**	70.60	39.77	-0.83	0.0279	immune response	saposin-like protein
**rhr-1**	98.18	55.23	-0.83	0.0047	ammonium metabolism	ammonium transporter family
**spp-5**	1,378.99	764.59	-0.85	0.0047	immune response	saposin-like protein
**ZK228.4**	54.29	29.96	-0.86	0.0047	immune response	innate immune response.

**Y51A2D.18**	12.07	6.57	-0.88	0.0199	transport	solute carrier family 22
**T13F3.6**	422.61	226.57	-0.90	0.0047	unknown	
**C24G6.6**	42.42	22.62	-0.91	0.0116	ammonium metabolism	polyamine oxidase (exo-N4-amino)) and SMOX (spermine oxidase); determine adult lifespan
**cyp-34A8**	17.68	9.34	-0.92	0.0047	oxidation-reduction process	cytochrome P450 family 21 subfamily A
**cyp-13A2**	7.62	4.02	-0.92	0.0376	oxidation-reduction process	cytochrome P450
**Y105C5B.15**	88.90	46.10	-0.95	0.0047	lipids metabolism	acid phosphatase
**21ur-15387,F55G11.4**	29.39	15.12	-0.96	0.0144	immune response	CUB-like domain
**amt-4**	19.80	10.06	-0.98	0.0047	ammonium metabolism	ammonium transporter protein family
**col-135**	22.79	11.25	-1.02	0.0047	structural protein/collagen/cuticle	Collagens
**clec-265**	29.31	14.44	-1.02	0.0047	unknown	C-type LECtin
**21ur-4206,21ur-4710,21ur-7217,ugt-22**	91.82	43.68	-1.07	0.0047	glucose metabolism	UDP glycosyltransferase family 3
**gst-13**	84.46	38.15	-1.15	0.0047	immune response	Glutathione S-transferase
**cah-4**	134.88	57.84	-1.22	0.0047	pH	carbonic anhydrase
**clec-66**	14.71	6.12	-1.27	0.0047	immune response	C-type LECtin
**dod-17**	7.59	3.14	-1.27	0.0116	determination of life span	epoxide hydrolase-1, determine adult lifespan
**cyp-35C1**	7.73	3.17	-1.29	0.0047	oxidation-reduction process	cytochrome P450s
**F28H7.3**	67.96	26.88	-1.34	0.0047	lipids metabolism	putative lipase domain
**clec-209**	21.89	8.48	-1.37	0.0047	immune response	C-type LECtin
**C33A12.19**	11.25	4.23	-1.41	0.0397	unknown	
**mtl-2**	672.05	251.21	-1.42	0.0047	metal stress	metallothionein
**F01D5.1**	13.85	5.10	-1.44	0.0174	immune response	innate immune response
**cyp-35A2**	27.75	10.09	-1.46	0.0047	lipids metabolism	cytochrome P450s
**C14C6.5**	135.81	44.87	-1.60	0.0047	immune response	Metridin-like ShK toxin
**clec-4**	9.90	3.24	-1.61	0.0047	immune response	C-type lectin
**F09C8.1**	5.05	1.57	-1.69	0.0047	lipids metabolism	phospholipase B1
**F01D5.5**	8.65	2.62	-1.73	0.0328	immune response	innate immune response
**F55G11.8**	6.28	1.87	-1.75	0.0047	immune response	epoxide hydrolase 1
**F01D5.3**	9.14	2.26	-2.01	0.0144	immune response	regulated by skn-1
**F35E12.5**	4.03	0.87	-2.21	0.0047	immune response	regulated by sbp-1 and elt-2
**dod-24**	43.00	8.84	-2.28	0.0047	immune response	epoxide hydrolase 1, determine adult lifespan
**F49F1.1**	3.96	0.72	-2.45	0.0047	immune response	dietary restriction down regulated
**C32H11.4**	5.77	1.04	-2.48	0.0047	immune response	epoxide hydrolase 1, innate immune response
**Y19D10B.7**	85.42	14.23	-2.59	0.0047	unknown	unknown
**mltn-10**	0.95	0.15	-2.70	0.0328	membrane	MLt-TeN related
**F15E11.15**	72.17	9.85	-2.87	0.0047	unknown	Up-regulated in Daf-2(gf)
**Y49G5A.1**	17.52	2.24	-2.97	0.0047	protease	serine-type endopeptidase inhibitor activity

**T24B8.5**	143.42	15.12	-3.25	0.0047	immune response	ShK-like toxin peptide
**cyp-35A3**	5.64	0.49	-3.53	0.0047	lipids metabolism	cytochrome P450s
**nspe-1**	19.27	1.00	-4.27	0.0116	unknown	Nematode Specific Peptide family, group E

List of genes regulated by glucose in the diet of wt (N2) worms (*q*-value <0.05). Worms were allowed to develop and grow on agar plates at 20°C until they reached stage L4, and then transferred to NGM plates with or without glucose and incubated for ~20 hours at 20°C.
Worms collected and RNA was isolated and subjected to RNA-seq according to Illumina guidelines. Differentially expressed genes were determined as described in Material and Methods. Genes are sorted by fold change of expression with respect to wt. *-information from www.wormbase.org.

Supplementary Table 2. Summary of all aging experiments.

Rep-eats	*C. elegans* strain	*E.coli* strain/ RNAi	Media/ treatment	Number of animals that died /total	50% survival, days	Mean survival, days ±SD	P-value, 1 2	Increase/decrease, %	Mean increase decrease ,% ±SD
1	N2	OP50	NGM	83/96	14.99	13.76±1.12			
2				69/100	12.8				
3				74/86	13.48				
1		OP50	NGM+ Glucose	63/98	10.32	9.52±1.00	0.00408	-31.15	-30.82 ±3.73
2				27/89	8.4			-34.38	
3				45/85	9.85			-26.93	
1	N2	OP50	NGM+ Diamide	37/75	17.39	17.19±0.44	0.00389	16.01	25.38 ±8.12
2				65/97	16.69			30.39	
3				88/97	17.49			29.75	
1		OP50	NGM+ Diamide+ Glucose	64/75	19.05	18.32±0.82	0.00233	27.08	33.47 ±5.55
2				82/98	17.44			36.25	
3				90/98	18.48			37.09	
1	N2	OP50	NGM+ Paraquat	66/92	18.5	17.18±1.87	0.03874	23.42	23.63 ±0.29
2				87/123	15.85			23.83	
3									
1		OP50	NGM+ Paraquat+ Glucose	76/99	14.77	14.04±0.65	0.36199	-1.47	2.29± 5.00
2				106/119	13.82			7.97	
3				81/100	13.53			0.37	
1	N2	OP50	NGM	107/126	12.62	12.45±0.50			
2				39/62	11.89				
3				92/119	12.84				
1		OP50	NGM+ Glucose	41/64	9.5	9.33±0.23	0.00029	-24.72	-24.98 ±4.26
2				60/102	9.41			-20.86	
3				82/122	9.07			-29.36	
1	N2	OP50	NGM+ Acet-aminophen	67/100	14.16	12.65±1.36	0.41259	12.2	1.53 ±9.28
2				118/151	11.54			-2.94	
3				102/138	12.24			-4.67	
1		OP50	NGM+Acet-aminophen +Glucose	62/120	11.35	11.25±0.09	0.00732	-10.06	-9.55 ±3.43
2				52/144	11.19			-5.89	
3				76/100	11.21			-12.69	
1	N2	HT115 + pL4440 RNAi	NGM	87/93	15.32	15.83±0.76			
2				81/100	16.7				
3				95/107	15.47				
1			NGM+ Glucose	92/96	12.54	12.53±1.24	0.00844	-18.15	-20.92 ±5.29
2				89/100	13.76			-17.61	
3				93/108	11.29			-27.02	
1	N2	HT115 + *gsr-1* RNAi	NGM	104/115	13.69	14.03±0.37	0.01043	-10.64	-11.26 ±4.87
2				90/100	13.96			-16.41	
3				78/80	14.43			-6.72	

1			NGM+ Glucose	97/105	11.22	10.68±0.66	0.00044	-26.76	-32.47 ±4.97
2				92/100	10.87			-34.91	
3				75/80	9.94			-35.75	
1	N2	HT115 + *gspd-1* RNAi	NGM	66/84	14.15	14.3±0.21	0.03819	-7.7	-10.59 ±4.08
2				77/80	14.45			-13.47	
3									
1			NGM+ Glucose	79/88	9.45	9.83±0.54	0.00123	-38.32	-38.59 ±0.38
2				81/85	10.21			-38.86	
3									
1	N2	HT115 + pL4440 RNAi	NGM	94/103	18.73	17.14±1.45			
2				83/100	16.82				
3				76/90	15.88				
1			NGM+ Glucose	87/100	11.71	12.56±0.77	0.00422	-37.48	-26.17 ±9.85
2				83/100	13.19			-21.58	
3				84/90	12.79			-19.46	
1		HT115 +*gsy-1* RNAi	NGM	76/101	19.69	19.66±0.3	0.02129	5.13	15.23 ±10.22
2				88/100	19.34			14.98	
3				74/90	19.94			25.57	
1			NGM+ Glucose	81/95	15.89	15.41±0.62	0.06468	-15.16	-9.78 ±7.17
2				95/100	14.71			-12.54	
3				90/100	15.62			-1.64	
1		HT115 +*pyg-1* T22F3.3 RNAi	NGM	97/100	18.48	18.03±1.49	0.25163	-1.33	5.7 ±13.41
2				85/93	16.36			-2.73	
3				87/98	19.24			21.16	
1			NGM+ Glucose	93/100	11.56	11.54±0.27	0.00138	-38.28	-32.32 ±6.32
2				87/95	11.27			-33.00	
3				90/100	11.8			-25.69	
1	N2	HT115 pL4440	NGM+ Diamide+Glucose	82/90	18.77	18.13±1.45			
2				92/100	19.16				
3				80/90	16.47				
1		HT115 *gsy-1*		70/90	18.89	18.41±0.71	0.38957	0.64	1.77± 4.61
2				62/80	18.75			-2.14	
3				79/80	17.6			6.86	
1		HT115 *pyg-1*		86/100	15.22	15.01±0.34	0.01113	-18.91	-16.96± 5.04
2				73/85	15.19			-20.72	
				79/90	14.62			-11.23	
1	*daf-2*	HT115 + pL4440 RNAi	NGM	84/90	34.61	34.83±0.35			
2				64/100	35.24				
3				79/125	34.65				
1			NGM+ Glucose	78/90	28.76	29.29±0.46	0.00004	-16.9	-15.91 ±1.16
2				63/97	29.53			-16.2	
3				85/115	29.58			-14.6	
1		HT115 +*gsy-1* RNAi	NGM	94/101	32.11	32.86±0.73	0.00678	-7.22	-5.66 ±2.22
2				82/106	32.9			-6.64	
3				109/156	33.57			-3.12	

1			NGM+ Glucose	99/107	31.51	32.95±1.31	0.03744	-8.96	-5.41 ±3.09
2				73/100	34.08			-3.29	
3				104/149	33.27			-3.98	
1		HT115 +*pyg-1* T22F3. 3 RNAi	NGM	83/102	32.92	37.34±3.86	0.16279	-4.88	7.15 ±10.43
2				74/99	40.04			13.62	
3				93/149	39.05			12.70	
1			NGM+ Glucose	85/110	21.36	21.66±1.03	0.00002	-38.28	-37.83 ±2.37
2				36/100	22.81			-35.27	
3				55/154	20.81			-39.94	
1	*daf-16*	HT115 + pL4440 RNAi	NGM	91/102	12.82	12.35±1.2			
2				96/100	10.99				
3				52/100	13.25				
1			NGM+ Glucose	47/100	10.1	9.55±0.49	0.01001	-21.21	-22.21 ±8.2
2				78/100	9.39			-14.56	
3				70/100	9.18			-30.88	
1		HT115 +*gsy-1* RNAi	NGM	73/106	14.9	13.95±0.88	0.06641	16.22	13.35 ±8.3
2				84/100	13.17			19.84	
3				50/100	13.83			4	
1			NGM+ Glucose	94/103	11.13	10.79±0.23	0.04679	-13.18	-12.17 ±8.16
2				63/100	10.6			-3.55	
3				37/100	10.64			-19.77	
1	*daf-2, daf-16*^	HT115 + pL4440 RNAi	NGM	86/105	14.45	13.15±1.24			
2				66/100	11.98				
3				86/100	13.03				
1			NGM+ Glucose	95/100	12.03	11.53±0.72	0.038671	-16.75	-12.24 ±0.26
2				60/100	10.55			-11.94	
3				86/100	12.1			-7.14	
				78/100	11.42			-13.16	
1		HT115 +*gsy-1* RNAi	NGM	108/110	15.81	14.56±1.16	0.112002	9.41	11.02 ±8.25
2				65/100	14.37			19.95	
3				76/100	13.51			3.68	
1			NGM+ Glucose	103/110	12.07	12.28±0.19	0.10455	-16.47	-6.18 ±8.50
2				59/100	12.48			4.17	
3				55/100	12.39			-4.91	
				86/100	12.16			-7.52	
1	*aak-2*	HT115 + pL4440 RNAi	NGM	62/80	12.64	12.76±0.16			
2				115/121	12.94				
3				63/80	12.71				
1			NGM+ Glucose	61/80	9.68	10.23±0.55	0.00076	-23.42	-19.85 ±3.35
2				109/112	10.77			-16.77	
3				68/80	10.25			-19.35	
1		HT115 +*gsy-1* RNAi	NGM	79/90	12.14	12.54±0.77	0.324580	-3.96	-1.79 ±4.87
2				96/100	13.43			3.79	
3				60/80	12.05			-5.19	
1			NGM+ Glucose	81/90	10.1	9.89±0.32	0.000075	-20.09	-22.53 ±2.5
2				95/100	10.04			-22.41	
3				58/80	9.52			-25.1	

1		OP50	NGM	88/102	14.83	14.24±1.23			
2				95/139	15.06				
3				37/90	12.82				
1	N2	OP50	NGM+ Glucose	85/120	11.19	10.14±1.03	0.00576	-24.54	-28.75 ±4.19
2				99/123	10.1			-32.93	
3				49/94	9.13			-28.78	
1		OP50	NGM+ 15 mM Diamide*	35/101	9.78	10.33±0.77			
2				43/73	10				
3				47/94	11.21				
1	N2*	OP50	NGM+15mM Diamide + Glucose*	51/115	12.13	12.72±0.75	0.00909	24.03	23.62 ±12.19
2				28/70	13.56			35.6	
3				49/112	12.47			11.24	
1		pL4440 RNAi	NGM	67/80	15.96	16.19±0.26			
2				60/80	16.14				
3				72/80	16.47				
1		*gsy-1* to pL4440	NGM	78/100	16.31	16.59±0.32	0.083675	2.19	2.46 ±2.31
2				90/100	16.93			4.89	
3				95/100	16.52			0.3	
1	N2	*gsy-1* RNAi	NGM	74/80	17.19	17.33±0.17	0.001516	7.71	7.04 ±1.88
2				73/80	17.51			8.49	
3				67/80	17.28			4.92	
1		pL4440 RNAi	NGM+ Glucose	71/80	11.73	11.79±0.12			
2				64/80	11.71				
3				70/80	11.92				
1		*gsy-1* to pL4440	NGM+ Glucose	88/100	13.25	13.05±0.61	0.01234	12.96	10.67 ±4.44
2				95/100	12.36			5.55	
3				74/100	13.53			13.51	
1		*gsy-1* RNAi	NGM+ Glucose	64/80	13.39	13.06±0.31	0.001315	14.15	10.78 ±2.92
2				47/80	12.78			9.14	
3				59/80	13			9.06	

Each data set (repeat) was fitted to a Boltzmann sigmoid curve and the mean survival time calculated. The % change in lifespan was with respect to the control in the same repeat. Independent experimental and control analyses, which were performed side-by-side, are indicated by the same number (1, 2, or 3) in the first column. Increase (+) or decrease (-) in lifespan is indicated. *p*-values were calculated with respect to control animals in the same experiment using Student's *t*-Test (one-tailed distribution and two-sample equal variance). *- worms tend to escape from plates with high concentration of diamide. ^ - Effect of *gsy-1* RNAi on *daf-2 daf-16* worms was at the border-line of statistical significance. However, in each individual experiment the *gsy-1* depletion increased the lifespan.

Supplementary Table 3. Summary of all experiments for glycogen measurements

Rep-eats	*C. elegans* strain and age	*E.coli* strain/ RNAi	Media/ treatment	Number of animals	Glycogen/ Protein, g/g	Mean glycogen content, g/g ±SD	change, %	Mean change, % ±SD
1	N2, A3	OP50	NGM	55	0.243	0.212±0.058		
2				45	0.248			
3				62	0.145			
1			NGM +Glucose	51	0.482	0.395±0.077	198.35	192.6 ±0.43
2				40	0.365		147.18	
3				59	0.337		232.41	
1			NGM +Diamide	80	0.0785	0.054±0.027	32.3	24.28 ±7.66
2				44	0.0583		23.51	
3				63	0.0247		17.03	
1			NGM +Diamide +Glucose	75	0.196	0.115±0.072	80.66	52.74 ±24.40
2				40	0.088		35.5	
3				56	0.061		42.07	
1	N2, A3	OP50	NGM	81	0.257	0.195±0.057		
2				48	0.146			
3				58	0.183			
1			NGM +Glucose	78	0.416	0.375±0.036	161.87	199.32 ±41.41
2				48	0.356		243.8	
3				58	0.352		192.3	
1			NGM+ Paraquate	78	0.1	0.081±0.038	38.9	52.65 ±17.68
2				91	0.106		72.6	
3				80	0.085		46.45	
1			NGM+ Paraquate+ Glucose	76	0.271	0.291±0.019	105.45	150.28 ±54.51
2				100	0.308		210.96	
3				80	0.246		134.43	
1	N2, A3	OP50	NGM	47	0.116	0.119±0.012		
2				46	0.11			
3				62	0.132			
1			NGM+ Glucose	50	0.167	0.196±0.038	143.97	163.49 ±18.62
2				43	0.182		165.45	
3				59	0.239		181.06	
1			NGM+ Acetamino-phen	50	0.09	0.115±0.033	77.59	97.93 ±34.86
2				98	0.152		138.18	
3				59	0.103		78.03	
1			NGM+Acet-aminophen+ Glucose	47	0.11	0.146±0.033	94.83	122.22 ±24.07
2				95	0.154		140	
3				58	0.174		131.82	
1	N2, A7	HT115 *pL4400*	NGM	32	0.296	0.369±0.118		
2				100	0.307			
3				48	0.505			
1			NGM+ Glucose	50	0.677	0.789±0.174	228.72	230 ±91.84
2				86	0.99		322.48	
3				47	0.701		138.81	
1		HT115 *gsy-1*	NGM	36	0.053	0.058±0.005	17.91	16.32 ±3.34
2				100	0.057		18.57	

3		RNAi		42	0.063		12.48	
1			NGM+ Glucose	41	0.072	0.098±0.034	24.32	28.72 ±14.22
2				95	0.137		44.62	
3				41	0.087		17.23	
1		HT115 *pyg-1* RNAi	NGM	43	2.436	2.151±0.411	823.5	639.1± 267.1
2				80	2.338		761.13	
3				71	1.68		332.83	
1			NGM+ Glucose	41	3.028	2.746±0.281	1023.65	801.78 ±278.9
2				67	2.743		893.07	
3				65	2.466		332.83	
1	N2, A7	HT115 *pL4400*	NGM+ Glucose +Diamide	62	0.259	0.354±0.083		
2				58	0.401			
3				71	0.404			
1		HT115 *gsy-1* RNAi		39	0.029	0.027±0.002		
2				47	0.025			
3				57	0.026			
1		HT115 *pyg-1* RNAi		61	1.29	1.498±0.242		
2				85	1.764			
3				54	1.439			
1	*daf-2*, A7	HT115 *pL4400*	NGM	68	0.62	0.732±0.192		
2				46	0.532			
3				40	0.819			
4				63	0.957			
1			NGM+ Glucose	60	0.87	1.01±0.29	140.32	136.81 ±11.92
2				38	0.662		124.44	
3				40	1.243		151.77	
4				53	1.251		130.72	
1		HT115 *gsy-1* RNAi	NGM	72	0.038	0.046±0.019	6.13	6.07 ±1.63
2				76	0.024		4.51	
3				20	0.068		8.3	
4				75	0.051		5.33	
1			NGM+ Glucose	63	0.050	0.056±0.009	8.06	7.77 ±0.82
2				82	0.046		8.65	
3				17	0.063		7.69	
4				78	0.064		6.69	
1		HT115 *pyg-1* RNAi	NGM	73	1.621	1.857±0.408	261.5	260.1 ±50
2				70	1.711		321.68	
3				22	1.632		199.31	
4				57	2.466		257.82	
1			NGM+ Glucose	74	1.837	2.296±0.615	296.4	329 ±121.9
2				69	2.655		499.19	
3				22	1.717		209.7	
4				71	2.974		310.88	

Glycogen amount was determined with a Glycogen Assay Kit (Sigma MAK016) and normalized to total protein content in the sample (For detailed procedure see Experimental Procedures). Independent experimental and control analyses, which were performed side-by-side, are indicated by the same number (1, 2, 3, or 4) in the first column. The percent in glycogen content was determined with respect to the control in the same repeat.

Supplementary Table 4. Summary of *sod-3*::GFP and *daf-2 sod-3*::GFP strains fluorescence intensity measurements

Ima-ges	*C. elegans* strain and age	*E.coli* strain/ RNAi	Media/ treatment	Number of animals	Intensity/ worm, AU	Intencity, %	Mean Intensity, %±SD	*p*-value
1	*sod-3*::GFP, A3	HT115 *pL4400*	NGM	5	1.8	100		
2				5	0.58	100		
3				5	0.3	100		
4				5	3.72	100		
5				5	0.48	100		
6				5	1.84	100		
7				5	1.52	100		
8				5	0.6	100		
9				5	0.6	100		
10				5	3.04	100		
1			NGM+ Glucose	5	1.14	63.33	54.31± 15.92	3.979 E-06 (compared to e.v. on NGM)
2				5	0.46	79.31		
3				5	0.16	53.33		
4				5	2.54	68.27		
5				5	0.18	37.5		
6				5	0.48	26.09		
7				5	0.98	64.47		
8				5	0.36	60		
9				5	0.28	46.67		
10				5	1.34	44.08		
1		HT115 *gsy-1* RNAi	NGM	5	3.14	174.44	247.17± 85.05	0.000197 (compared to e.v. on NGM)
2				5	1.86	320.69		
3				5	1.12	373.33		
4				5	6.98	187.63		
5				5	1.82	379.17		
6				5	3.76	204.35		
7				5	4	263.16		
8				5	0.86	143.33		
9				5	1.48	246.67		
10				5	5.44	178.95		
1			NGM+ Glucose	5	3.1	172.22	215.81± 65.27	1.261E-05 (compared to e.v. on NGM)<br><br>0.183954 (compared to *gsy-1* on NGM)
2				5	1.5	258.62		
3				5	0.84	280		
4				5	4.9	131.72		
5				5	1.5	312.5		
6				5	2.96	160.87		
7				5	3.12	205.26		
8				5	1.4	233.33		
9				5	1.64	273.33		
10				5	3.96	130.26		
1	*daf-2 sod-3*::GFP, A3	HT115 *pL4400*	NGM	5	1.32	100		
2				5	1.12	100		
3				5	1.24	100		
4				5	1.58	100		
5				5	2.3	100		

6				5	1.34	100		
7				5	1.32	100		
8				5	0.94	100		
9				5	0.94	100		
10				5	1.28	100		
11				5	1.1	100		
12				5	1.38	100		
13				5	1.18	100		
1			NGM+ Glucose	5	0.88	66.67	82.01± 11.17	3.2902E-07 (compared to e.v. on NGM)
2				5	0.66	58.93		
3				5	1.06	85.48		
4				5	1.22	77.22		
5				5	1.82	70.14		
6				5	0.94	81.82		
7				5	1.08	82.98		
8				5	0.78	95.74		
9				5	0.9	87.5		
10				5	1.12	90.91		
11				5	1	84.06		
12				5	1.16	96.61		
13				5	1.14	88		
1		HT115 *gsy-1* RNAi	NGM	5	2.12	160.61	170.33± 37.13	6.999E-07 (compared to e.v. on NGM)
2				5	1.98	176.79		
3				5	1.94	156.45		
4				5	2.38	150.63		
5				5	2.32	155.24		
6				5	2.08	139.39		
7				5	1.84	242.55		
8				5	2.28	236.17		
9				5	2.22	207.81		
10				5	2.66	176.36		
11				5	1.94	121.74		
12				5	1.68	149.15		
13				5	1.76	141.33		
1			NGM+ Glucose	5	1.4	106.06	147.6± 36.73	7.1244E-05 (compared to e.v. on NGM)<br><br>0.089915 (compared to *gsy-1* on NGM)
2				5	1.36	121.43		
3				5	1.64	132.26		
4				5	1.93	122.15		
5				5	2.01	140.3		
6				5	1.88	169.7		
7				5	2.24	221.28		
8				5	2.08	202.13		
9				5	1.9	178.13		
10				5	2.28	163.64		
11				5	1.8	133.33		
12				5	1.84	127.12		
13				5	1.5	101.33		

Fluorescence intensity was quantified using Zeiss ZEN software package. For each image five worms per condition were grouped together. The % of intensity was calculated with respect to the control in the same image. *p*-values were calculated in Microsoft Excel using Student's *t*-Test (one-tailed distribution and two-sample equal variance).

Supplementary Table 5. List of SiRNA sequences for glycogen synthase knockdown in hepatocytes.

Name	Length	Sequence
siGYS2.1		
siGYS2.1-SEQ1	25	rGrUrC rArCrU rUrUrU rGrArA rArUrC rUrArG rArArU rUrGA T
siGYS2.1-SEQ2	27	rArUrC rArArU rUrCrU rArGrA rUrUrU rCrArA rArArG rUrGrA rCrArU
siGYS2.2		
siGYS2.2-SEQ1	25	rGrCrA rArUrC rArGrC rUrGrA rCrUrA rArGrU rUrUrC rUrCT A
siGYS2.2-SEQ2	27	rUrArG rArGrA rArArC rUrUrA rGrUrC rArGrC rUrGrA rUrUrG rCrArA
siGYS2.3		
siGYS2.3-SEQ1	25	rGrGrA rCrUrU rUrUrC rArArC rArArC rCrGrC rArCrA rGrAT A
siGYS2.3-SEQ2	27	rUrArU rCrUrG rUrGrC rGrGrU rUrGrU rUrGrA rArArA rGrUrC rCrArA
